# Analysis of Ku70 S155 Phospho-Specific BioID2 Interactome Identifies Ku Association with TRIP12 in Response to DNA Damage

**DOI:** 10.3390/ijms24087041

**Published:** 2023-04-11

**Authors:** Sanna Abbasi, Laila Bayat, Caroline Schild-Poulter

**Affiliations:** 1 Department of Biochemistry, Schulich School of Medicine and Dentistry, Western University, London, ON N6A 3K7, Canada; sabbasi5@uwo.ca (S.A.); lbayat@uwo.ca (L.B.); 2 Robarts Research Institute, Schulich School of Medicine and Dentistry, Western University, London, ON N6A 5B7, Canada

**Keywords:** Ku70, TRIP12, proximity-dependent biotin identification (BioID2), phosphorylation, double-stranded breaks, proximity ligation assay

## Abstract

The Ku heterodimer, composed of subunits Ku70 and Ku80, is known for its essential role in repairing double-stranded DNA breaks via non-homologous end joining (NHEJ). We previously identified Ku70 S155 as a novel phosphorylation site within the von Willebrand A-like (vWA) domain of Ku70 and documented an altered DNA damage response in cells expressing a Ku70 S155D phosphomimetic mutant. Here, we conducted proximity-dependent biotin identification (BioID2) screening using wild-type Ku70, Ku70 S155D mutant, and Ku70 with a phosphoablative substitution (S155A) to identify Ku70 S155D-specific candidate proteins that may rely on this phosphorylation event. Using the BioID2 screen with multiple filtering approaches, we compared the protein interactor candidate lists for Ku70 S155D and S155A. TRIP12 was exclusive to the Ku70 S155D list, considered a high confidence interactor based on SAINTexpress analysis, and appeared in all three biological replicates of the Ku70 S155D-BioID2 mass spectrometry results. Using proximity ligation assays (PLA), we demonstrated a significantly increased association between Ku70 S155D-HA and TRIP12 compared to wild-type Ku70-HA cells. In addition, we were able to demonstrate a robust PLA signal between endogenous Ku70 and TRIP12 in the presence of double-stranded DNA breaks. Finally, co-immunoprecipitation analyses showed an enhanced interaction between TRIP12 and Ku70 upon treatment with ionizing radiation, suggesting a direct or indirect association in response to DNA damage. Altogether, these results suggest an association between Ku70 phospho-S155 and TRIP12.

## 1. Introduction

In response to DNA damage, a network of signaling pathways collectively referred to as the DNA damage response (DDR) is initiated. In the case of double-stranded breaks, once such damage has been detected, the activation of the Ataxia-telangiectasia mutated (ATM) kinase leads to the phosphorylation of numerous targets, including CHK2, 53BP1, BRCA1, p53, and histone H2A.X at serine 139 (also referred to as γ-H2A.X), which accumulates at DSBs [1]. These events subsequently lead to cell cycle checkpoint arrest, allowing the cell time to assess and repair the damage or trigger apoptosis if the damage is too great [2]. Over the years, multiple proteins have been implicated in both DNA repair and the DDR, with some being explored as potential cancer therapeutic targets [3].

Previous work in our laboratory has implicated the Ku complex, well-established for its role as a double-stranded break (DSB) sensor and scaffold protein, in the DDR. Within the Ku70 von Willebrand A (vWA) domain, work in our laboratory previously identified serine 155 (S155) as a novel phosphorylation site that was phosphorylated in response to severe ionizing radiation (IR) [4,5]. To study the effect of the Ku70 S155 residue, two mutants were used: S155A, a phosphoablative substitution which loses phosphorylation potential at this site, and S155D, a phosphomimetic substitution that mimics constitutive phosphorylation [4,5]. 

After IR treatment, Ku70^−/−^ mouse embryonic fibroblast (MEF) cells expressing each mutant displayed contrasting effects in terms of cell survival. Ku70 S155D-expressing cells displayed decreased cell survival, whereas Ku70 S155A-expressing cells displayed prolonged survival despite having normal DNA repair activity [4,5]. Notably, even in the absence of DNA damage, expression of Ku70 S155D alone was shown to result in specific characteristics, including the altered expression of certain cell cycle and apoptosis regulatory genes, cell cycle arrest with most cells arrested in the G1 and G2 phases, and decreased cell proliferation [5]. Our laboratory has previously shown that Ku70 S155A and S155D substitutions do not impact DNA repair activity [4,5]. These results imply that the Ku70 S155 mutations do not affect Ku70 binding to core NHEJ factors and that this residue may be important for functions outside of DSB repair. Intriguingly, expression of the Ku70 vWA domain with the S155D mutation alone was capable of inciting the same phenotype as the full-length protein, implying that the Ku70 vWA domain, which cannot bind Ku80 or double-stranded DNA, is sufficient to elicit the observed effects. The effect of Ku70 S155D was also previously demonstrated to be dominant over wild-type Ku70 in human IMR90 cells [5].

Altogether, these findings led to the working hypothesis that the phosphorylation of Ku70 S155 may function to promote and/or sustain a DDR at DSBs. The working model proposed that Ku70 is localized to DSBs and upon a cellular assessment of the DNA damage, if the damage was deemed overwhelming for repair, Ku70 S155 was then phosphorylated [5]. Exactly how Ku70 S155 phosphorylation fits into the order of DDR events is still unclear [5].

The DDR pathway is intimately connected to the DNA repair pathway as crosstalk between the two pathways coordinates the cellular response to damage [1,6]. While Ku70 S155D expression appeared to correlate with changes in gene expression and cell cycle progression, it was unclear how this post-translational modification was promoting such changes. We previously hypothesized that the phosphorylation of Ku70 S155 could be promoting a potentially novel protein interaction that could be leading to the observed effects [5]. Using a pull-down assay with Ku70 S155D, work in our laboratory previously identified Aurora B, a mitotic kinase, as a candidate protein that interacted not only with Ku70 S155D, but also with Ku70 following IR treatment, implying an interaction with Ku70 phospho-S155 [5]. However, it was unclear whether the interaction between Ku70 S155D or Ku70 phospho-S155 and Aurora B was direct and whether this interaction was, on its own, responsible for the effects of Ku70 S155 phosphorylation [5]. Therefore, in the research conducted here, we expanded our screening approach to identify in vivo Ku70 S155D-specific interactors in human cells. Since the interaction with Ku70 S155D could have also been transient or weak in nature, a more expansive approach was needed to identify all possible candidates in an unbiased manner. To identify such candidates, we employed the proximity-based in vivo tagging technique, BioID2 [7].

As previously hypothesized, we postulate here that the phosphorylation of Ku70 S155 initiates a key protein–protein interaction with Ku70 that leads to the observed phenotypes. Consequently, it is expected that the protein interaction(s) will be absent in Ku70 S155A cells, which cannot be phosphorylated. To our knowledge, this is the first instance of BioID2 being used to identify phospho-specific protein interactor candidates.

## 2. Results

### 2.1. Establishing Flp-In^TM^ T-REx 293 BioID2 Stable Cell Lines

**Figure 1:**
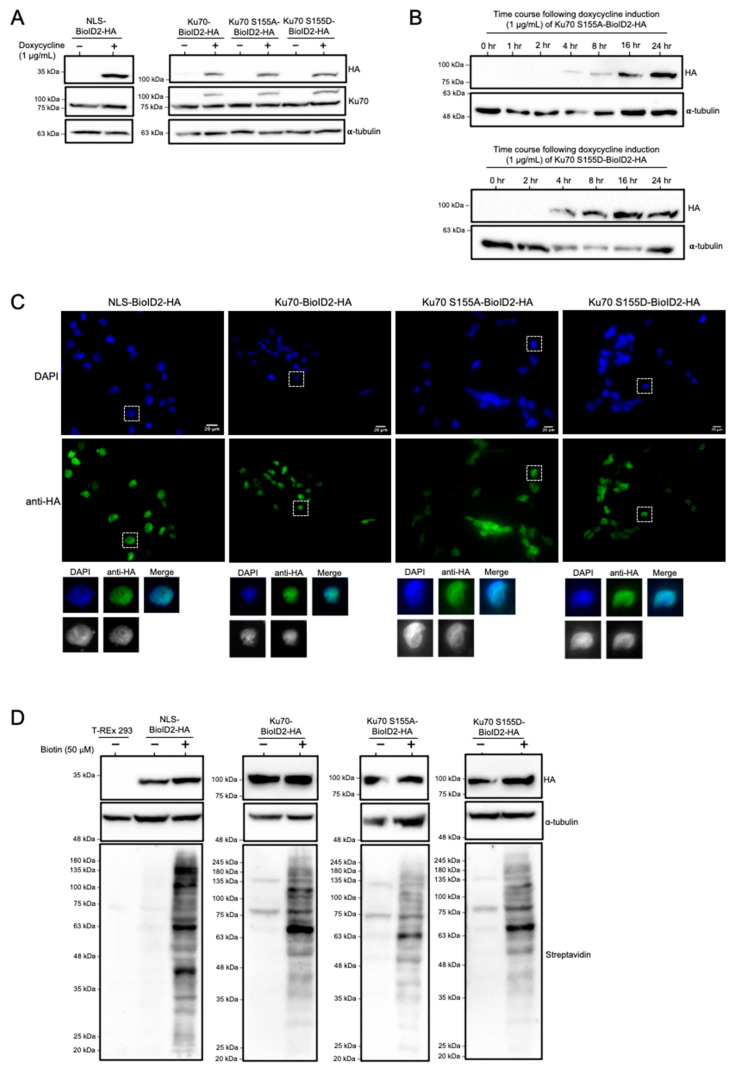
Creating and testing inducible, stable Ku70-BioID2 Flp-In^TM^ T-REx 293 cell lines. All Western blots depicted show 50 µg of whole cell extracts (WCE). Western blots were probed with HA, Ku70, and α-tubulin antibodies, and a HRP-streptavidin probe: (**A**) cell lines induced with or without 1 µg/mL doxycycline for 24 h before analyzing by Western blotting; (**B**) time course experiment to determine optimal expression time point of stable cell lines induced with 1 µg/mL doxycycline using Ku70 S155A-BioID2-HA and Ku70 S155D-BioID2-HA stable cell lines; (**C**) indirect immunofluorescence of BioID2 T-REx 293 stables cell lines induced with 1 µg/mL doxycycline for 24 h, using HA antibody, imaged at 488 nm wavelength. Images were taken at 20× magnification and the white line denotes 20 µm. Below, cropped enlargements of individual cells are shown either colored or in grey-scale; (**D**) Biotinylation of doxycycline (1 µg/mL)-induced stable cell lines. After 8 h of doxycycline induction, cell lines were supplemented with or without biotin (50 µM) for 24 h prior to analysis by Western blotting.

To identify candidates that could be associated with Ku70 S155D using BioID2, we first established stable cell lines expressing the Ku70-BioID2 biotin ligase fusion proteins. As Ku70 S155D had previously demonstrated a dominant phenotype that led to checkpoint activation and cell cycle arrest [4,5], it was necessary to use a regulatable promoter to prevent the constitutive expression of Ku70 S155D. The Flp-In^TM^ 293 T-REx^TM^ pcDNA.5/FRT/TO inducible Tet-ON expression system was used to create stable, inducible cell lines, whereby the Ku70-BioID2 fusion protein (wild-type, Ku70 S155A, or Ku70 S155D) or the Ku70 NLS-BioID2 fusion protein was stably integrated in the T-REx 293 cell line and under the control of a doxycycline-inducible promoter (Figure 1A). As a control, we used the Ku70 S155A mutant, making the assumption that the protein(s) we were looking for would not associate with the phosphoablative mutant. The Ku70 NLS-BioID2 and the parental T-REx 293 cell line (without any BioID2 fusion proteins) were used as subsequent negative controls to determine non-specific binding proteins for BioID2 screening (Figure 1A).

As the BioID2 technique necessitates a 16–24 h incubation period with biotin [7], to determine the optimal time to induce expression prior to biotin incubation, a time course experiment was conducted for both T-REx 293 Ku70 S155A- and S155D-BioID2 cell lines (Figure 1B). For both cell lines, only faint expression was detectable by immunoblotting after 4 h, thus 8 h post-induction was selected as the point at which to add biotin to the media for biotin incubation in subsequent BioID2 experiments (Figure 1B).

In addition to testing expression and optimizing induction conditions, fusion protein localization was also tested using indirect immunofluorescence with the stable cell lines. As expected, the vast majority of cells demonstrated consistent nuclear localization and the results also showed that most cells expressed the fusion proteins (Figure 1C). Our previous work with Ku70 using both BioID [8] and BioID2 [9] indicated that a C-terminal addition of the biotin ligase would not hinder Ku70 nuclear localization, function, or heterodimerization with Ku80.

Finally, the biotinylation of the BioID2 fusion proteins was also tested, as described previously [8,9], and each of the BioID2 fusion proteins demonstrated robust biotinylation 24 h after 8 h of doxycycline induction, and only with the addition of supplemental biotin (Figure 1D).

### 2.2. Conducting BioID2: Filtering and Selecting Final Ku70 S155D-Specific Candidate Interactor Proteins

**Figure 2:**
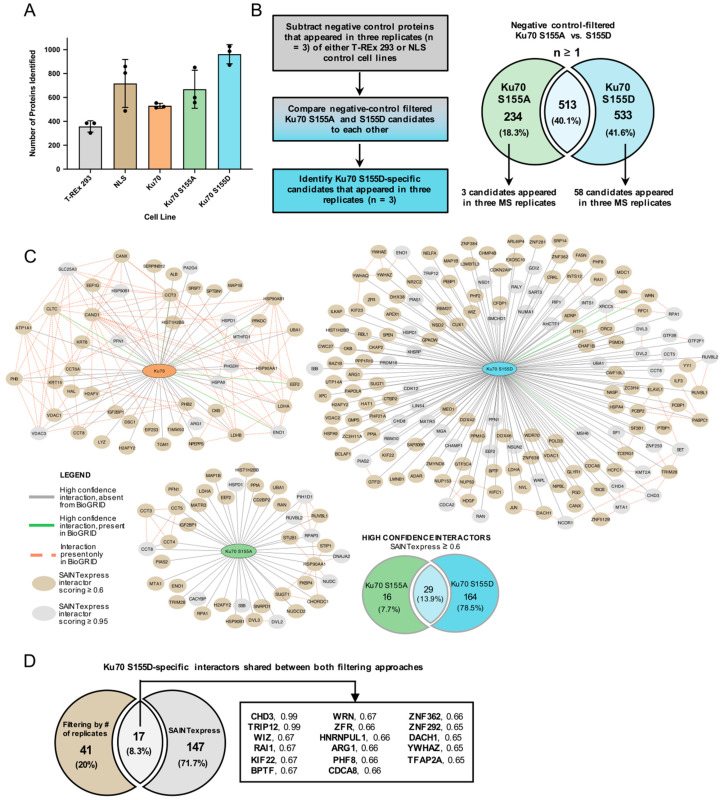
BioID2 candidate filtering and comparison process for final selection of Ku70 S155D-specific interactors: (**A**) total number of proteins identified in three biological replicates of BioID2 in Flp-In^TM^ T-REx 293 stable cell lines: parental T-REx 293, NLS (Ku70)-BioID2, Ku70 WT-BioID2, Ku70 S155A-BioID2, and Ku70 S155D-BioID2; (**B**) flow chart of the filtering process used to select final candidates prioritizing number of replicates. Venn diagram compares negative control-filtered Ku70 S155A- and S155D-BioID2 proteins appearing in one or more (*n* ≥ 1) MS replicates before focusing on those exclusive to Ku70 S155D that appeared in three biological replicates; (**C**) quantitative SAINTexpress analysis to determine high confidence interactors for wild-type Ku70, Ku70 S155A, and Ku70 S155D before comparing Ku70 S155A and S155D candidates with a SAINTexpress score of ≥ 0.6 and focusing on those exclusive to Ku70 S155D; (**D**) final seventeen Ku70 S155D-specific candidates determined combining both qualitative and quantitative filtering approaches with a SAINTexpress score indicated beside each candidate.

Three biological replicates of BioID2 were conducted for two negative control cell lines: parental T-REx 293 cells and NLS-BioID2, and for the three experimental BioID2 cell lines: wild-type Ku70, Ku70 S155A, and Ku70 S155D (Figure 2A). As determined earlier, BioID2 cell lines were induced for 8 h with doxycycline before supplemental biotin was added for 24 h. On average, 357 proteins were identified for T-REx 293 cells, 717 proteins were identified for NLS-BioID2 cells, 530 proteins for wild-type Ku70-BioID2 cells, 669 proteins for Ku70 S155A-BioID2, and 962 proteins for Ku70 S155D-BioID2 cells (Figure 2A).

Two different approaches were used to filter and select the final candidate proteins. In the first approach, the non-specific proteins that appeared in three replicates of either negative control cell line (Table S1), T-REx 293 and/or NLS-BioID2, were subtracted from the Ku70 S155A-BioID2 and Ku70 S155D-BioID2 protein candidate lists. Next, the negative control-filtered lists were compared to each other to identify Ku70 S155D-exclusive candidate proteins, focusing only on those that appeared consistently in all three biological MS replicates (Figure 2B). Fifty-eight proteins were identified for Ku70 S155D while only three candidates remained for Ku70 S155A (Table S2).

In the second approach, a more quantitative method was used to identify candidate interactors from the BioID2 data. Here, we used SAINTexpress (Significance of Analysis of INTeractome) [10], a computational algorithm specifically designed to determine “high confidence” interactors primarily using quantitative data (in this case, protein spectral counts) to compare the protein candidates identified in an experimental cell line to those identified in control cell lines. SAINTexpress was conducted for wild-type Ku70-BioID2, Ku70 S155A-BioID2, and Ku70 S155D-BioID2 data, with each being compared to the proteins identified for both negative control cells (Figure 2C). SAINTexpress interactors scoring 0.6 or more (Table S3) were compared between Ku70 S155A-BioID2 and Ku70 S155D-BioID2, and 16 proteins were found to be specific to Ku70 S155A while 164 were found to be specific to Ku70 S155D (Figure 2C). In addition, the SAINTexpress candidates were also compared between the three experimental cell lines: wild-type Ku70, Ku70 S155A, and Ku70 S155D (Figure S1). The Ku70 S155D candidates identified by SAINTexpress (193 candidates, scoring 0.6 or more) were further analyzed using GO to determine which biological processes were significantly overrepresented (Figure S2).

To determine the final lists of Ku70 S155-specific candidate proteins, the Ku70 S155D-specific candidates identified using both approaches described above were compared, and 17 proteins were found to be shared (Figure 2D). For Ku70 S155A-specific candidates, only two candidates were found to be shared: RPAP3 and NUDCD2, likely due to the fact that only three candidates remained following the first filtering approach. As we hypothesize that phosphorylation of Ku70 S155 promotes a protein interaction and since Aurora B had previously been identified as a Ku70 phospho-S155-specific interactor [5], we focused our validation efforts on the Ku70 S155D-specific interactor candidates. The SAINTexpress score, indicated beside each candidate (Figure 2D), was used to decide which candidates to test for association with Ku70 S155D. We focused on TRIP12, which achieved a high SAINTexpress score of 0.99, for validation testing.

### 2.3. Establishing Flp-In^TM^ T-REx HeLa Ku70-HA Stable Cell Lines

**Figure 3:**
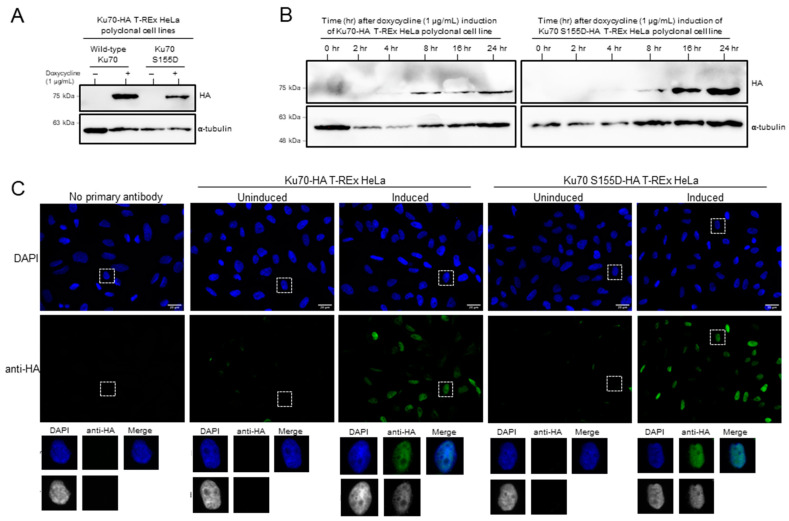
Generating doxycycline-inducible wild-type Ku70-HA and Ku70 S155D-HA Flp-In^TM^ T-REx HeLa stable cell lines. All Western blots depicted show 50 μg of whole cell extracts (WCE), with or without doxycycline (1 μg/mL). Western blots were probed with HA and α-tubulin antibodies: (**A**) Western blot of inducible stable cell lines with and without 24 h of doxycycline; (**B**) time course experiment showing the inducible expression of both polyclonal stable cell lines induced with doxycycline over a period of 24 h; (**C**) cell lines induced with or without doxycycline for 24 h before analysis by indirect immunofluorescence using HA antibody, imaged at 647 nm wavelength. Images were taken at 40× magnification with the white line representing 20 μm. Representative images are shown with cropped enlargements of individual cells colored (top) or grey-scaled (below).

To validate the selected candidate interactor for association with Ku70 S155D, two doxycycline-inducible polyclonal stable cell lines were created using the Flp-In^TM^ T-REx HeLa expression system: wild-type Ku70-HA and Ku70 S155D-HA (Figure 3A). Unlike the previously established Flp-In^TM^ T-REx 293 stable lines, these constructs did not contain the BioID2 biotin ligase addition. Rather than using T-REx 293 cells again, T-REx HeLa cells were specifically chosen as they would be better for visualizing in situ interactions due to their larger cell and nuclei size.

Both stable cell lines demonstrated expression after 8 h of doxycycline induction and greater expression after 24 h of doxycycline induction (Figure 3B). Additionally, both cell lines were also tested for Ku70-HA expression and localization using indirect immunofluorescence and both demonstrated expression of Ku70-HA in the majority of doxycycline-induced cells and confirmed that their expression was nuclear (Figure 3C).

### 2.4. Testing BioID2 Candidate TRIP12 for Co-Localization with Ku70 S155D-HA

As was performed by previous studies for the validation of BioID/BioID2 candidates [9,11,12,13,14], PLA was selected as the validation method of choice for the BioID2 candidate interactor. To validate the selected candidate interactor, TRIP12, for its association with Ku70 S155D specifically, PLA was conducted using both the T-REx HeLa wild-type Ku70-HA and Ku70 S155D-HA doxycycline-inducible stable cell lines, where cells were either induced or not induced with doxycycline. We also included an analysis of a Ku70 S155A-HA doxycycline-inducible stable cell line created with the same T-REx HeLa expression system (Figure S3). If TRIP12 was a true Ku70 S155D-specific interactor/proximal protein, it would be expected to show a PLA co-localization signal with induced Ku70 S155D-HA, but not with wild-type Ku70-HA or Ku70 S155A-HA.

**Figure 4:**
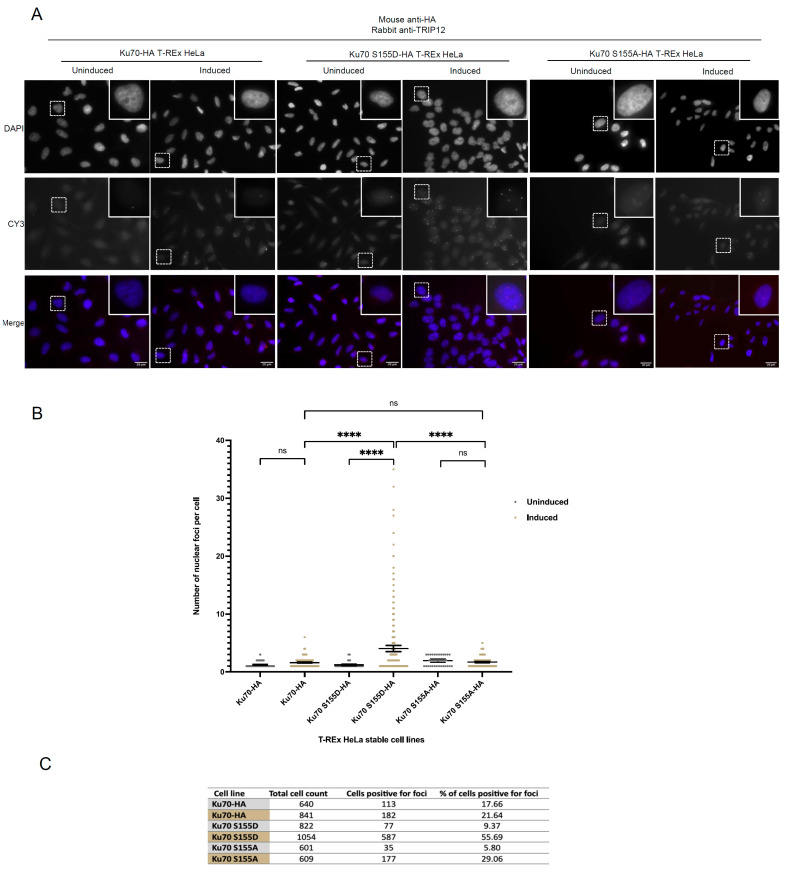
PLA analysis with wild-type Ku70-HA, Ku70 S155D-HA, and Ku70 S155A-HA T-REx HeLa stable cell lines and candidate protein TRIP12: (**A**) wild-type Ku70-HA, Ku70 S155D-HA, and Ku70 S155A-HA stable cell lines were induced with doxycycline (1 μg/mL) for 24 h prior to seeding and fixing cells, then PLA was conducted using mouse MINUS and rabbit PLUS probes to determine if there was an association between Ku70 S155D-HA and the Ku70 S155D-specific BioID2 candidate protein TRIP12. Images were taken at 40× magnification with the white line representing 20 μm. Insets show enlargements of individual cells; (**B**) PLA quantification using ImageJ. Average number of nuclear foci determined for a minimum of 600 cells from 1–2 technical replicates (different cell passages) from four separate PLA experiments (biological replicates); only cells positive for foci are plotted. The bars represent the mean with 95% confidence intervals indicated. The “ns” stands for not significant, while **** indicates a *p*-value less than 0.0001; (**C**) proportion of cells positive for foci per cell line. The number of cells counted from four separate biological replicates is shown with the number and proportion of cells positive for foci.

For the candidate protein TRIP12, representative PLA images are depicted (Figure 4A) and the number of nuclear foci observed in each cell line were counted using ImageJ to determine the average number of nuclear PLA foci per cell, representing co-localization events between Ku70-HA and TRIP12 (Figure 4B,C).

### 2.5. Testing for TRIP12 and Ku70 Association in the Presence of DSBs

**Figure 5:**
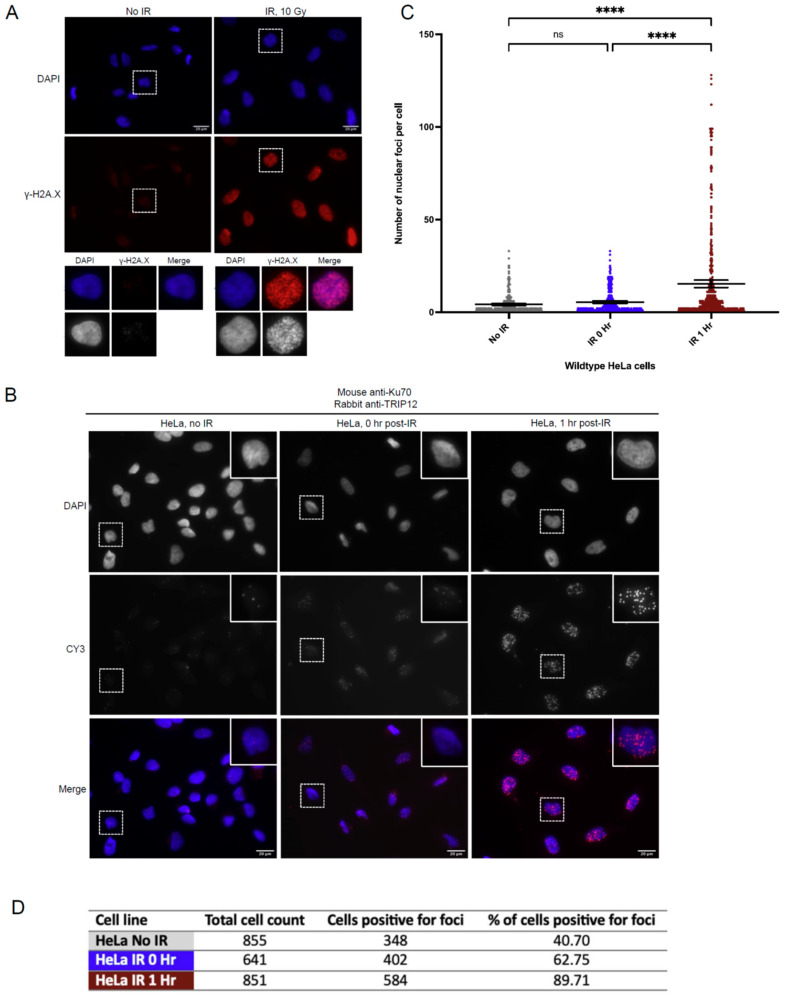
PLA with endogenous Ku70 and TRIP12 in HeLa cells treated with IR to produce DSBs: (**A**) indirect immunofluorescence testing HeLa cells treated with 10 Gy IR for the presence of DSBs using a specific antibody to detect γ-H2AX. Images were taken at 40× magnification. Below, cropped enlargements of individual cells are shown either colored or in grey-scale; (**B**) representative images of PLA conducted with endogenous Ku70 and TRIP12 in HeLa cells without IR (no IR), with 10 Gy IR and fixed immediately (IR 0 h), or with cells fixed 1 h post-10 Gy IR (IR 1 h), visualized with immunofluorescence microscopy. Images were taken at 40× magnification with the white line representing 20 μm. Insets show enlargements of individual cells; (**C**) PLA quantification of the number of nuclear foci per cell was determined using ImageJ. The average number of nuclear foci are depicted as bar graphs and calculated for a minimum total of 600 cells for each condition from four technical replicates for each of three separate experiments or biological replicates; only cells positive for foci are plotted. The bars represent the mean with 95% confidence intervals indicated. The “ns” stands for not significant, while **** indicates a *p*-value less than 0.0001; (**D**) proportion of cells positive for foci. The number of cells counted from three separate biological replicates is shown with the number and the proportion of cells per treatment that were positive for foci.

Since TRIP12 demonstrated a robust PLA localization signal with the induced Ku70 S155D-HA stable cell line and is well-known for its role as a DDR protein, we wanted to test whether the association between endogenous TRIP12 and Ku70 was present after treating cells with ionizing radiation (IR), which introduces DSBs. Previously, Fell et al. (2016) were able to demonstrate that Ku70 was phosphorylated at S155 in response to severe IR treatment, and they hypothesized that Ku70 S155 was being recruited and then subsequently phosphorylated at DSBs [5]. We aimed to replicate these conditions to test if the selected candidate, TRIP12, would show an altered interaction following IR treatment. PLA was conducted in HeLa cells with endogenous Ku70 and TRIP12. HeLa cells treated with 10 Gy IR were checked, via indirect immunofluorescence, for the presence of DSBs using an antibody recognizing phospho-H2A.X at serine residue 139 (γ-H2A.X), which can visualize the accumulation of γ-H2A.X at DSBs (Figure 5A).

Three separate biological replicates, each including four technical replicates, of PLA were conducted in HeLa cells with the following three conditions: no IR, IR 0 hr (cells were treated with 10 Gy IR and fixed immediately), or IR 1 hr (IR-treated cells were fixed after 1 h to allow for complete protein recruitment to DSB breaks) (Figure 5B). We evaluated the percentage of cells positive for foci for the three conditions (Figure 5C,D). In untreated cells (No IR), we found that approximately 41% of cells had at least one focus, while 63% were positive for foci in the IR 0 h condition, and 90% were positive for foci in the IR 1 h condition (Figure 5D). PLA results were then quantified to determine the average number of nuclear foci per cell in cells positive for foci. On average, approximately four PLA nuclear foci were detected for endogenous Ku70 and TRIP12 in untreated HeLa cells, approximately five nuclear foci in IR 0 h cells, while an average of fifteen nuclear foci were observed for HeLa cells 1 h after IR treatment (Figure 5C). There was no significant difference between the no IR and IR 0 h condition (adjusted *p*-value = 0.0612); however, there were significantly more foci on average in the IR 1 h condition compared to both no IR (adjusted *p*-value < 0.0001) and the IR 0 h (adjusted *p*-value < 0.0001) conditions (Figure 5C). 

**Figure 6:**
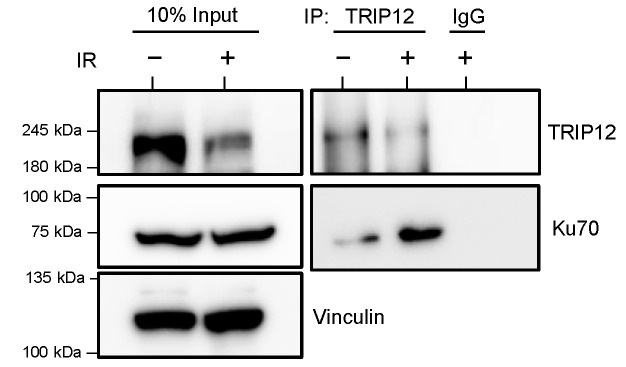
Ku70 interaction with TRIP12 is enhanced in response to DNA damage. HeLa cells were left untreated (−) or subjected to 40 Gy of IR (+) and whole cell extracts were prepared after 1 h incubation. Immunoprecipitation was performed (*n* = 3) with a TRIP12 antibody (or control IgG) and immunoprecipitates were analyzed with antibodies Ku70 and TRIP12. Equal loading of inputs was assessed with a vinculin antibody.

Finally, to determine complex formation between Ku and TRIP12 in response to DNA damage, we performed co-immunoprecipitation experiments. Ku was found to weakly co-immunoprecipitate with TRIP12 in non-treated HeLa cells, and this was greatly enhanced upon treatment of cells with IR (Figure 6). This analysis confirms that the strong association detected by PLA is due to an interaction (direct or indirect) between Ku and TRIP12 following DNA damage.

Altogether, these data demonstrate that TRIP12 and Ku interact in response to DNA damage, providing support to the idea that DNA damage-dependent Ku70 S155 phosphorylation may facilitate TRIP12 association.

## 3. Discussion

The Ku70 S155 residue, first identified in our laboratory as a novel phosphorylation site, was previously implicated in the DDR [4,5]. Exactly how Ku70 phospho-S155 exerts its effects are unknown, though we hypothesized that the phosphorylation of Ku70 S155 could be promoting protein–protein interaction(s). Here, we conducted a BioID2 screen comparing the phosphomimetic mutant, Ku70 S155D, to the phosphoablative mutant, Ku70 S155A, to identify Ku70 S155D-specific interactor candidate proteins. Using two different filtering approaches, including a quantitative SAINTexpress analysis, we identified several Ku70 S155D-specific candidates and selected TRIP12 for further validation testing. Using an inducible expression system with HA-tagged wild-type Ku70, Ku70 S155A, and Ku70 S155D, we conducted PLA experiments with the candidate protein to determine if TRIP12 co-localized with Ku70 S155D specifically. TRIP12 demonstrated a robust PLA signal with induced Ku70 S155D, validating its identification as a Ku70 S155D-specific proximal interactor. Finally, through co-immunoprecipitation and PLA, we demonstrated complex formation and co-localization between Ku70 and TRIP12 1 h after IR treatment, suggesting Ku70 and TRIP12 associate in response to DNA damage.

Both the BioID and BioID2 techniques have traditionally been used with full-length, wild-type bait proteins [7,8,11,13,15]. Recent studies have attempted to expand upon these techniques and use them in a non-traditional manner; for example, by identifying residue- or domain-specific interactors [8,16,17]. The study described here is the first, to our knowledge, to use BioID2 to identify phospho-dependent interactor candidates.

Phosphorylation is a well-studied post-translational modification, documented to promote changes in cellular localization and translocation, signal transduction, or temporary protein interactors [18]. Using a Ku70 S155D pull-down, Fell et al. (2016) were able to identify a phospho-dependent protein interaction between Ku70 S155 and Aurora B, and this led us to suspect that Ku70 phospho-S155 may function by mediating a protein interaction. In this current work, we did not observe Aurora B in our Ku70 S155D-BioID2 mass spectrometry results. However, we did find Borealin (CDCA8, Figure 2D), which is a known member of the INCENP complex. The INCENP complex consists of Borealin, Aurora B, and Survivin [19]. At this point, it is unclear exactly how or why Ku70 may be interacting with the INCENP complex, but it is possible that the biotin ligase was unable to efficiently tag the other members due to conformational constraints.

Protein interactions can be highly diverse. Some of the most well studied protein interactions are constitutive and strong, capable of being co-immunoprecipitated with a bait protein of interest [20]. Such interactions are preserved under in vitro conditions and are capable of withstanding mild to harsh binding conditions. On the other hand, weaker and transient protein interactions are more difficult to study and validate [20]. Fortunately, various techniques have been developed to identify and/or study weak and transient protein interactions in vivo, including the likes of fluorescence resonance energy transfer (FRET) microscopy [21], PLA [22], and BioID/BioID2 [7,15]. 

In this study, we chose to employ the BioID2 screening technique, which is capable of identifying, theoretically, all types of protein interactions (e.g., weak, transient, strong, proximal, etc.). BioID2 was deemed a suitable and more encompassing technique as we wanted to identify additional Ku70 phospho-S155-specific factors that could have been missed in the previous Ku70 S155D pull-down [5]. In addition, using BioID2, we wanted to gain insight into the in vivo protein environment of the Ku70 S155D mutation, that is, identifying the proteins that may be in proximity to Ku70 S155D. GO analysis of the Ku70 S155D-BioID2 candidates showed multiple biological processes being significantly enriched. Some of the overrepresented pathways were not unexpected, including processes involving DNA DSB repair and telomere-related functions. Others were more unexpected, such as viral expression and mitosis-related processes. For example, mitotic sister chromatid separation was one biological process enriched in the Ku70 S155D cells.

Thyroid hormone receptor-interacting protein 12 or TRIP12, is an E3 ubiquitin ligase previously implicated in the DDR for its regulation of other proteins involved directly or indirectly at DSBs [23,24,25]. TRIP12 showed a robust PLA signal in most induced Ku70 S155D-HA cells, but not with wild-type Ku70-expressing cells (Figure 4B), suggesting a specific interaction with the phosphomimetic form of Ku70 S155. A recent study has found that while TRIP12 mRNA levels remain constant, TRIP12 protein expression appears to vary based on cell cycle phase [26], providing one explanation as to why not all cells seem to show PLA signal. A second explanation could also be that not all cells expressed Ku70-HA in the inducible T-Rex HeLa stable cell lines. As stated earlier, PLA can be used to verify proximity-based interactions, and from our results, we can establish that TRIP12 is found in proximity to HA-tagged Ku70 S155D, the phosphomimetic substitution representing Ku70 phospho-S155.

To determine a context for when TRIP12 and Ku70 phospho-S155 could be interacting, we tested for a PLA association between TRIP12 and Ku70 in the presence of DSBs. Previously, Fell et al. (2016) were able to demonstrate that Ku70 S155 is phosphorylated 30 min after IR treatment, compared to untreated cells. Our PLA results with cells fixed immediately after IR (0 h IR) and cells fixed 1 h after IR (1 h IR) seem to suggest that endogenous Ku70 and TRIP12 only significantly co-localize with one another 1 h after IR treatment (Figure 5C). These results suggest their interplay is not part of an immediate response, but that the proteins may be in close proximity at complex or more severe DSBs where DNA damage may require prolonged assessment/repair or signaling of an unsuccessful repair attempt [27]. Depending on the complexity of the break or the extent of damage, DSBs can take anywhere between 30 min to several hours to be resolved [28]. Fell et al. (2016) previously hypothesized that the phosphorylation of Ku70 S155 could be occurring at complex DSBs, for which repair may take more time to be completed, or in response to overwhelming DNA damage (e.g., ≥10 Gy IR), which may not be able to undergo repair at all [5].

Based on the previous model and our current preliminary results, Ku70 S155 is phosphorylated in response to DNA damage, and the co-localization between Ku70 and TRIP12 could be dependent on this phosphorylation. Since TRIP12 has previously been involved in modulating the DDR through limiting the activity of RNF168 at DNA breaks [25], the interplay between Ku and TRIP12 could be involved in the DDR and/or DNA repair. 

While we have been able to establish a proximity-based association between TRIP12 and Ku70 S155D and an indirect/direct TRIP12 association with Ku70 in response to DNA damage, the context of this association needs further investigation. One possibility is that TRIP12 co-localization with Ku at DSBs promotes either activation or inhibition of its E3 activity. Alternatively, phosphorylation of Ku70 S155 could be a signal triggering its ubiquitination via TRIP12. Although we have yet to establish whether TRIP12 acts on Ku through its E3 ligase activity or whether Ku functions to modulate TRIP12 activity in response to DNA damage, there are other functional contexts that may explain this proximity-based interaction. For example, although TRIP12 is currently best known for its role as an E3 ubiquitin ligase acting on specific substrates at DSBs, TRIP12 has also recently been implicated in mitotic cell cycle progression and chromosome stability from experiments using TRIP12-depleted HeLaS3 cells [26], offering additional functional contexts to consider. 

To our knowledge, this research was the first to use the BioID2 technique to screen for phospho-specific interactor candidates. In summary, we established inducible expression systems to conduct both the BioID2 screening and the validation testing. We were able to validate TRIP12 as a novel Ku70 S155D-specific interactor and provided a potential context for a TRIP12-Ku70 phospho-S155 interaction at DSBs.

## 4. Materials and Methods

### 4.1. Mammalian Expression Constructs

Previously, site-directed mutagenesis was used to introduce substitution mutations S155A and S155D within wild-type Ku70 (XRCC6) cDNA [4]. Wild-type Ku70-BioID2 was previously described [9], and Ku70 S155A and Ku70 S155D cDNAs were likewise cloned into the BioID2 plasmid (Addgene) by PCR amplification with the same primers: Ku70 full-length (fl) BioID HpaI forward primer (5′-ATCGTGGTTAACCGGATGTCAGGGTGGGAGTCATATTACA-AAACCGAG) and Ku70 full-length (fl) BioID BamHI reverse primer (5′-TACGATGGATCCCGCGCAGTCCTGGAAGTGCTTGGTGAGGGC), using the same restriction enzyme cloning strategy [9]. Ku70 NLS-BioID2 plasmid was also previously described [9].

All four BioID2 cloning experiments were subsequently used to sub-clone the NLS-BioID2-HA or Ku70-BioID2-HA fusion constructs (wild-type, S155A, and S155D) into the Flp-In^TM^ T-Rex^TM^ expression system plasmid, pcDNA.5/FRT/TO (ThermoFisher Scientific, Rockford, IL, USA) using a blunt–blunt restriction enzyme cloning strategy. The NLS-BioID2-HA and Ku70-BioID2-HA constructs were excised from the BioID2 plasmid by digestion with HpaI and PmeI and gel-purified prior to ligation with the EcoRV-digested pcDNA.5/FRT/TO plasmid. All cloning was verified by sequencing analysis, conducted by the DNA Sequencing Facility at Robarts Research Institute, London, ON, Canada.

For the validation testing, doxycycline-inducible Ku70-HA, Ku70 S155D-HA, and Ku70 S155A-HA stable cell lines were created. Wild-type Ku70-HA was created and then subsequently subcloned into the EcoRV site of the pcDNA.5/FRT/TO plasmid. The Ku70 S155D and A mutations were subcloned into wild-type Ku70-HA pcDNA.5/FRT/TO using BamHI and EcoRV restriction enzyme sites. The N-terminal portion of Ku70 containing the S155D or S155A mutation was PCR-amplified using the previously cloned Ku70 S155D/A-BioID2 plasmids (described earlier) as a template with primers: Ku70 Fwd BamHI (TTAATAGGATCCATGTCAGGGTGGGAGTCATATTACAAAACC) and Ku70 Rev EcoRV (TTAATAGATATCTCTGTAGAACAAGGATATGTCAAAGCCCCC), which introduced BamHI and EcoRV restriction sites to the ends of the PCR product.

### 4.2. Cell Culture and Reagents

The following human cell lines were used in this study: HeLa (purchased from ATCC, Manassas, VA, USA), Flp-In^TM^ T-Rex^TM^ 293 doxycycline-inducible cells (ThermoFisher Scientific), and Flp-In^TM^ T-Rex^TM^ HeLa doxycycline-inducible cells (generated by Dr. Arshad Desai’s laboratory, San Diego, CA, USA). All cell lines were cultured in high-glucose Dulbecco’s modified Eagle’s medium (DMEM) (Wisent Bioproducts, St. Bruno, QC, Canada) supplemented with 10% fetal bovine serum (FBS) (Wisent Bioproducts) at 37 °C in 5% CO_2_. For biotinylation, cells were incubated for 24 h with media supplemented with 50 μM biotin at 37 °C in 5% CO_2_. Prior to doxycycline-inducible stable cell line creation, Flp-In^TM^ T-Rex 293 and T-Rex HeLa cell lines were maintained in 15 μg/mL blasticidin (Wisent Bioproducts) and 100 μg/mL zeocin (Invitrogen, Life Technologies, Burlington, ON, Canada), as necessitated by the manufacturer to maintain integration of the FRT site and Tet repressor protein, respectively. After generation of the stable doxycycline-inducible cell lines, both T-Rex^TM^ 293 and T-Rex^TM^ HeLa cells were maintained in 15 μg/mL blasticidin and 150 μg/mL hygromycin (Wisent Bioproducts). To induce expression, 1 μg/mL doxycycline was added to the media for 24 h, or less for time course experiments.

Doxycycline-inducible Flp-In^TM^ T-Rex^TM^ 293 and T-Rex HeLa cell lines were used to create doxycycline-inducible stable cell lines expressing NLS-BioID-HA and Ku70-BioID2-HA (wild-type, S155A, and S155D) or wild-type Ku70-HA, Ku70 S155D-HA, and Ku70 S155A-HA, respectively. Stable cell lines were created by following manufacturer’s directions (ThermoFisher Scientific). Briefly, cloned pcDNA.5/FRT/TO plasmid constructs were co-transfected with pOG44 plasmid containing the Flp recombinase enzyme at a 1:9 ratio, respectively, before selection with 150 μg/mL hygromycin. Co-transfections were conducted using jetPRIME transfection reagent (Polyplus-transfection, Illkirch-Graffenstaden, France), following manufacturer instructions. Following successful integration events, monoclonal cells could be observed growing after approximately 10 days and were pooled and screened for expression by indirect immunofluorescence and Western blotting.

### 4.3. Ionizing Radiation (IR) Treatments

For immunofluorescence analyses, HeLa cells were seeded onto 24-well coverslips and the next day cells were irradiated with 10 Gy IR in an X-ray cell irradiator cabinet (CellRad™, Precision X-ray Inc., North Branford, CT, USA). Coverslips were either immediately fixed afterward and processed for PLA or incubated at 37 °C in 5% CO_2_ for 1 h prior to fixing and processing for PLA (below). For the co-immunoprecipitation analyses, cells were seeded onto 15 cm plates, irradiated with 40 Gy of IR, and incubated for 1 h followed by collection and extract preparation.

### 4.4. Protein Extracts, Western Blots, and Co-Immunoprecipitation

Whole cell extracts (WCE) were prepared either using WCE buffer or RIPA lysis buffer, as previously described [8]. Protein extracts were resolved by SDS-PAGE (8% or 10%) before transferring onto a polyvinylidene difluoride (PVDF) membrane and blocking in 5% skim milk in TBST solution. PVDF membranes were hybridized overnight with mouse anti-HA (H3663, Sigma-Aldrich, Oakville, ON, Canada, 1:1000) and mouse anti-α-tubulin (T5168, Sigma-Aldrich, 1:1000) primary antibodies. Similarly, biotinylated proteins could also be detected by immunoblotting, as described previously [9]. Western blots were imaged using the Clarity Western ECL substrate (Bio-Rad, Hercules, CA, USA) and the Molecular Imager^®^ ChemiDoc^TM^ XRS system (Bio-Rad) with Image Lab (v6.0.1).

For the Ku70 co-immunoprecipitation, wild-type HeLa cells untreated or treated with IR were collected to produce 500 µg of WCE that was diluted to 0.01% NP-40 and pre-cleared for 30 min, followed by a 2 h incubation at 4 °C with an anti-TRIP12 antibody (A301-814A, Bethyl, Waltham, MA, USA). Immunoprecipitated proteins were bound using Pierce^TM^ Protein G magnetic beads (ThermoFisher Scientific) and washed five times in wash buffer (20 mM HEPES pH 7.4, 100 mM NaCl, 0.5 mM EDTA, 0.05% NP-40, 12% glycerol) before being analyzed by Western blotting.

### 4.5. Indirect Immunofluorescence

Indirect immunofluorescence was conducted as described previously [9]. Briefly, seeded cells were fixed, permeabilized, blocked, then incubated overnight at 4 °C with primary mouse antibodies: HA (H9658, Sigma-Aldrich, Oakville, ON, Canada, 1:1000) or γ-H2A.X (ab26350, abcam, UK 1:1000). The next day, cells were washed and then incubated for 1 h in the dark with secondary antibody mouse Alexa 647 (Invitrogen, Life Technologies, Burlington, ON, Canada, 1:1000). Coverslips were mounted with ProLong Gold containing 4′,6′-diamidino-2-phenylindole (DAPI) (Invitrogen, Life Technologies). Images were taken at 40× magnification with an Olympus BX51 microscope using Image-Pro Plus (v5.0) software (Media Cybernetics, Inc., Bethesda, MD, USA). All indirect immunofluorescence figures were generated using ImageJ (v2.1.0) and zoomed-in; single-cell panels were generated using the QuickFigures plug-in [29].

### 4.6. Proximity-Dependent Biotin Identification (BioID2) and Mass Spectrometry Analysis

BioID2 was conducted exactly as previously described [9]. Samples were prepared as previously described for mass spectrometry [9]. For all MS samples, the missed cleavage rate was on average 6.22%, with the highest missed cleavage rate being 7.84%, while the lowest was 4.25%.

Samples were quantified and processed as previously described [9] before submission to the UWO Biological Mass Spectrometry Laboratory/Dr. Don Rix Protein Identification Facility (London, ON, Canada) for an analysis of peptides by high-resolution LC-ESI-MS/MS. The samples were run on an ACQUITY M-Class UHPLC system (Waters, Milford, MA, USA) connected to an Orbitrap Elite mass spectrometer (ThermoFisher Scientific). As described previously [9], the solution composition was as follows: Solution A consisted of water/0.1% formic acid (FA) while Solution B was acetonitrile (ACN)/0.1% FA. Peptides (~1 μg) were injected onto an ACQUITY UPLC M-Class Symmetry C18 Trap Column, 5 μm, 180 μm × 20 mm, and trapped for 6 min at a flow rate of 4 μL/min at 99% Solution A/1% Solution B. Peptides were separated on an ACQUITY UPLC M-class Peptide BEH C18 Column, 130 Å, 1.7 μm, 75 μm × 250 mm, operating at a flow rate of 300 nL/min at 35 °C using a non-linear gradient consisting of 1–7% Solution B over 1 min, 7–23% Solution B over 179 min and 23–35% Solution B over 60 min before increasing to 95% Solution B and washing. Samples were run in positive ion mode.

Data acquisition and parameters were the same as previously described [9]. To reiterate, data were acquired using an FT/IT/CID Top 20 scheme with lock mass and were processed using PEAKS Studio version 8.5 (Bioinformatics Solutions Inc., Waterloo, ON, Canada), using 3 missed cleavages and semi-specific cleavage. Parent mass error tolerance was set to 20 ppm, while fragment mass error tolerance was set to 0.8 Da. Protein and peptide false discovery rate (FDR) was set to 1%. Cysteine carbamidomethylation was set as a fixed modification while oxidation (M) and N-terminal deamidation (NQ) were set as variable modifications (maximum number of modifications per peptide = 5). All raw MS files were searched in PEAKS Studio version 8.5 using the Human Uniprot database (reviewed only; updated November 2019). For result filtration parameters, proteins were identified using a minimum of ≥1 unique peptide(s) with the FDR set to 1%. The mass spectrometry proteomics data have been deposited to the ProteomeXchange Consortium [30] via the PRIDE [31] partner repository with the dataset identifier PXD025581 and 10.6019/PXD025581.

### 4.7. Filtering and Selecting BioID2 Candidates for Validation

Scaffold v4.8.7 (Proteome Software Inc., Portland, OR, USA) was used to validate the MS/MS-based peptide and protein identifications, based on the Peptide Prophet algorithm [32] with Scaffold delta-mass correction and the Protein Prophet algorithm [33], respectively. Peptides had to be identified with at least 95% probability, and only those proteins identified with a minimum 95% probability (resulting in a protein FDR < 1%) using at least two unique peptides were analyzed further. Spectral counts were exported from Scaffold for all stable cell lines and formatted for SAINTexpress analysis [10] (available at https://reprint-apms.org; accessed on 14 March 2021). Proteins with a SAINTexpress score ≥0.6 were incorporated into a network figure, highlighting the high confidence interactors scoring ≥0.6 and ≥0.95. Protein network mapping information was visualized using Cytoscape (v3.6.1).

### 4.8. Ku70 S155D Candidate Analysis Using Gene Ontology (GO)

GO enrichment analysis by biological process was conducted for the 193 Ku70 S155D BioID2 candidate proteins with a SAINTexpress score of ≥0.6. A selection of significantly enriched biological processes (*p* < 0.05, using Bonferroni correction for multiple testing) with a fold-change ≥5 are depicted in Figure S2.

### 4.9. Duolink^®^ In Situ Proximity Ligation Assay (PLA)

PLA was conducted as described previously [9]. Cells were incubated at 4 °C overnight with mouse and rabbit antibodies. The primary mouse antibodies used were against either HA (H9658, Sigma-Aldrich, 1:500) or Ku70 (N3H10, Santa Cruz, 1:1000). Rabbit primary antibodies used were for Ku80 (H-300, Santa Cruz, 1:1000) and TRIP12 (HPA036835, Sigma-Aldrich, 1:800. Following primary incubation, PLA was conducted by following the manufacturer’s instructions (Sigma-Aldrich). Probes used were anti-Mouse MINUS and anti-Rabbit PLUS. Cells were mounted as described for immunofluorescence and images were taken with an Olympus BX51 microscope at 40× magnification (using the CY3 channel) and Image-Pro Plus (v5.0) software (Media Cybernetics, Inc.). All PLA figures were generated using ImageJ (v2.1.0) and zoomed-in, single-cell panels were generated using the QuickFigures plug-in [29].

Quantitative analysis to determine the number of nuclear PLA puncta or foci per cell was performed using ImageJ (v2.1.0). Binary DAPI images were used to highlight each nucleus (size set to 0.003-infinity) as a ROI. Similarly, binary images outlining the PLA foci (size set to 0.0003-infinity) were also acquired and merged with the DAPI outline.

For each of the T-REX HeLa stable cell lines, a minimum of 600 cells were randomly counted in total from four biological replicates of PLA (cells were induced, fixed, and processed on different days). Of those 600, only cells with one or more nuclear foci (cells positive for foci) were counted to determine the average number of nuclear foci per cell for each cell line, and these values were plotted as bars.

Similarly, for the HeLa cells PLA, a minimum of 600 cells were also counted for all conditions (no IR, IR 0 h, and IR 1 h) from three biological PLA replicates, each including exactly four technical replicates (cells were induced, fixed, and processed on the same day). Of those cells, only cells with one or more nuclear foci were counted to determine the average number of nuclear foci per cell for each cell line.

For both PLA analyses, the bar indicates mean with 95% confidence intervals. Statistics were performed on GraphPad Prism 8.0 using a one-way ANOVA with a Sidak’s multiple comparisons test, where **** indicates *p* ≤ 0.0001.

## Data Availability

The mass spectrometry data detailed in this manuscript have been submitted to the ProteomeXchange Consortium via the PRIDE partner repository with the dataset identifier PXD025581 and 10.6019/PXD025581.

## Supplementary Materials

The following supporting information can be downloaded at: https://www.mdpi.com/article/10.3390/ijms24087041/s1.
