# Supplementary material for: Analysis of Ku70 S155 Phospho-Specific BioID2 Interactome Identifies Ku Association with TRIP12 in Response to DNA Damage
Source: Int J Mol Sci. 2023 Apr 11;24(8):7041. doi: 10.3390/ijms24087041 (PMC10138931; doi:10.3390/ijms24087041)

SUPPLEMENTARY

VDAC3
MTHFD1
ATP5F1B
PA2G4
PHGDH
SLC25A3
HSP90AB1
H2AC4
HAL
TGM1
SERPINB12
DSC1
PHB
H2AFV
TIMM50
LDHB

KRT15
KRT8
NPEPPS
ALB
LYZ
CAND1
ATP1A1
EEF1G
CLTC
EIF2S3
SRSF7
RPL36A
SPTBN1
CCT6A
TUBA1B

HSP90B1
IGF2BP1

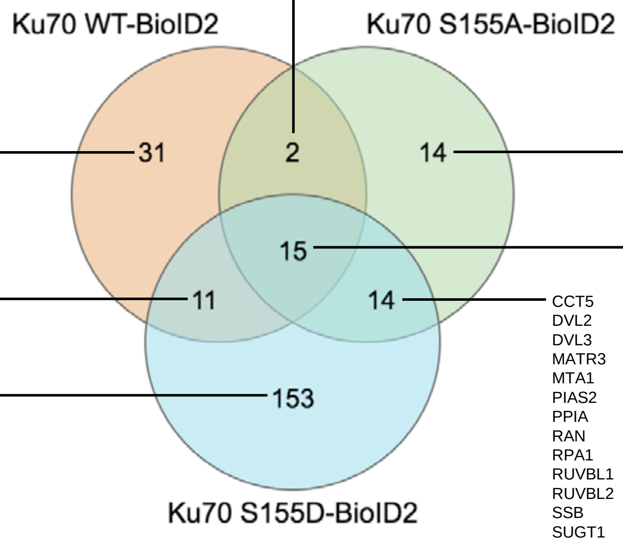

RPAP3
NUDC
PIH1D1
CACYBP
DNAJA2
CHORDC1
FKBP4
STIP1
NUDCD2
TUBA1A
CCT4
STUB1
SNRPD1
CD2BP2

CCT3
CCT8
EEF2
ENO1
H2AFY2
H2BC4
HIST1H2BB
HSP90AA1
HSPD1
LDHA
MAP1B
PFN1
RPS18
UBA1
XRCC6

CCT5
DVL2
DVL3
MATR3
MTA1
PIAS2
PPIA
RAN
RPA1
RUVBL1
RUVBL2
SSB
SUGT1
TRIM28

ARG1
CANX
CKB
HNRNPM
HSPA9
PHB2
PRKDC
RPL27A
RPS19
RPS4X
VDAC1

TRIP12
GTF2B
CHAMP1
MGA
RALY
CDCA2
SF1
KHSRP
XRCC5
INTS1
MSH6
KMT2A
SART3
LIN54
NUMA1
ZNF250
GDI2
RBM10
RIF1
CDK12
NSD1
NSUN2
CHD3
CHD4
PRDM16

WAPL
PIAS1
CHD8
GTF2F1
AHCTF1
SMCHD1
SET
PCBP2
DDX42
BAZ1B
RTF1
MDC1
PCBP1
BCLAF1
VDAC2
DDX46
CDKN2AIP
UTP14A
RPL19
ZNF638
PHF2
ZNF512B
NSD2
TCERG1
NUP50

SAP30BP
KIFC1
RFC1
ZC3H4
MED1
CFDP1
RPS27A
INTS12
PTBP1
GPKOW
NBN
ELAVL1
ADNP
ILKAP
CWC27
PEBP1
ZC3H11A
PPM1G
EXOSC10
DHX38
ILF3
WRN
GTF2I
ZNF384
NR2C2

RAI1
NASP
BPTF
KIF23
KIF22
HDGF
YY1
NUP153
RBL1
WIZ
HCFC1
HSPA4
NELFA
CTBP2
PPP1R10
TBCB
PGD
XPC
CDCA8
FASN
RPS3A
YWHAQ
PAPOLA
ZFR
ZMYND8

JUN
LMNB1
CUX1
RPS23
APEX1
NIPBL
PHF8
ZNF362
PABPC1
PHF21A
SRP14
RBM27
HNRNPUL1
ORC2
ADAR
ANP32A
ZNF281
GTF3C4
PSMD4
TCEA1
CHAF1B
WDR70
GMPS
POLD3
DIDO1

HN1L
YWHAZ
YWHAE
SPEN
GLYR1
ATP5PO
RPLP0P6
TFAP2A
CKAP2
ZNF292
PIAS3
DACH1
HAT1
PRDX4
NVL
RPS2
CWF19L1
CHMP4B
PKM
SF3B1
GNB2L1
RPS24
HSPA5
CRKL
L3MBTL3

ARL6IP4
SREK1
NCOR1

Figure S1. Three-way Venn diagram of wild-type Ku70-BioID2, Ku70 S155A-BioID2, and Ku70 S155D-BioID2 SAINTexpress protein candidates. Comparison was conducted with candidate proteins with a SAINTexpress score of ≥ 0.6 (candidate proteins listed for each cell line listed in Supplementary Table S3).

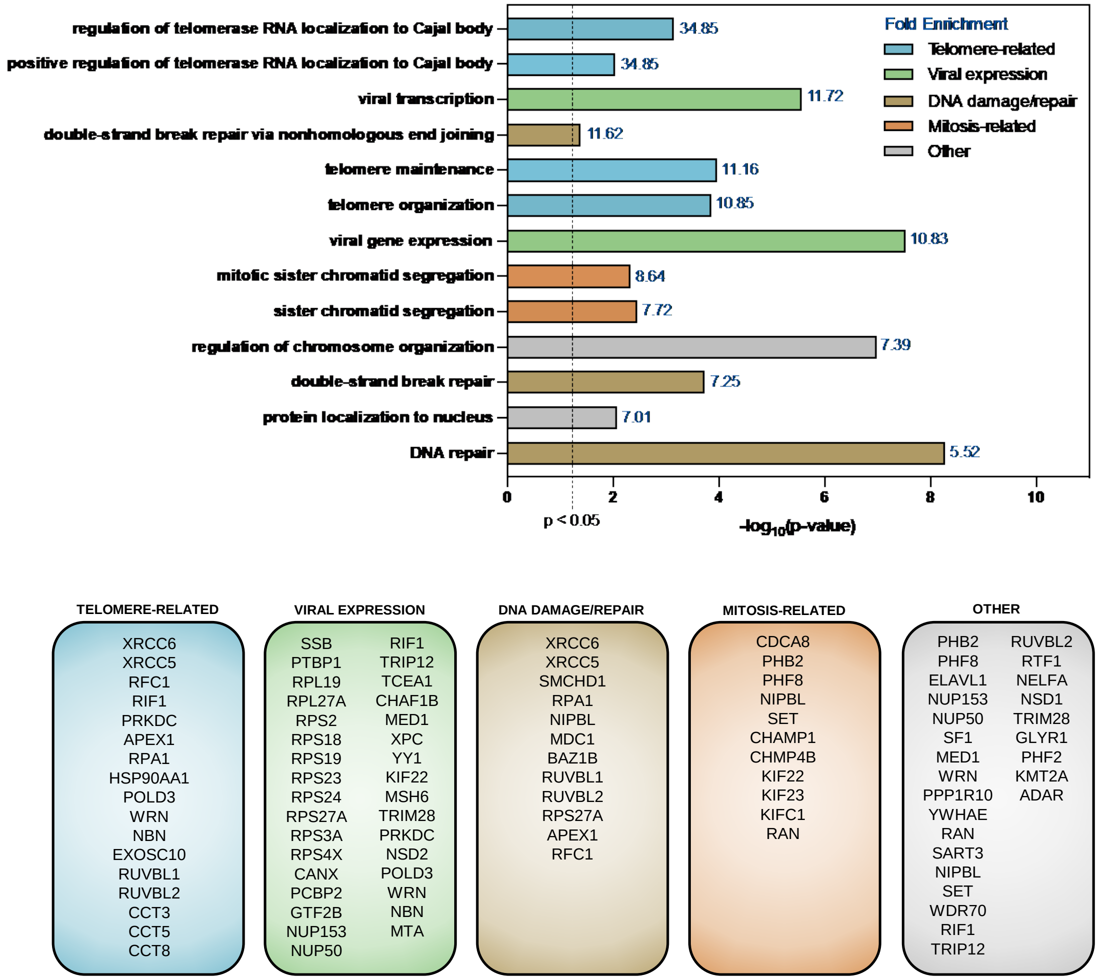

Figure S2. Gene Ontology (GO) analysis of Ku70 S155D BioID2 candidates. GO enrichment analysis (Fisher's Exact test) by complete biological process was conducted for the 193 Ku70 S155D BioID2 candidate proteins with a SAINTexpress score of ≥ 0.6*;* bar graph depicts a selection of significantly enriched biological processes ($p < 0.05$, using Bonferroni correction for multiple testing) with a fold-change ≥ 5. Candidate proteins, indicated by gene name, identified for each grouping of GO biological processes.

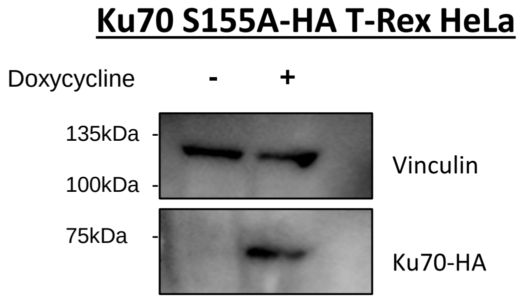

Figure S3. Induction of Ku70 S155A-HA T-Rex HeLa stable cell lines with or without 1 µg/mL doxycycline for 24 hours before analyzing by Western blotting. Blots were probed with HA and vinculin antibodies.

Supplementary Table S1A. Proteins appearing in three MS replicates of negative control – T-REx 293

Gene Name	Detailed Description
ACACA	Acetyl-CoA carboxylase 1
PC	Pyruvate carboxylase, mitochondrial
PCCA	Propionyl-CoA carboxylase alpha chain, mitochondrial
MCCC1	Methylcrotonoyl-CoA carboxylase subunit alpha, mitochondrial
KRT10	Keratin, type I cytoskeletal 10
MYH9	Myosin-9
KRT1	Keratin, type II cytoskeletal 1
KRT9	Keratin, type I cytoskeletal 9
MYH10	Myosin-10
KRT2	Keratin, type II cytoskeletal 2 epidermal
ACACB	Acetyl-CoA carboxylase 2
RSL1D1	Ribosomal L1 domain-containing protein 1
NOP56	Nucleolar protein 56
KRT5	Keratin, type II cytoskeletal 5
TUBB	Tubulin beta chain
NOP2	Putative ribosomal RNA methyltransferase NOP2
KRT14	Keratin, type I cytoskeletal 14
HIST1H1C	Histone H1.2
KNOP1	Lysine-rich nucleolar protein 1
TUBB4B	Tubulin beta-4B chain
HIST1H1E	Histone H1.4
HSPA8	Heat shock cognate 71 kDa protein
NOP58	Nucleolar protein 58
H2AFY	Core histone macro-H2A.1
TOP1	DNA topoisomerase 1
HSPA1A	Heat shock 70 kDa protein 1A/1B
TCOF1	Treacle protein
ATP5A1	ATP synthase subunit alpha, mitochondrial
KRT16	Keratin, type I cytoskeletal 16
RPL6	60S ribosomal protein L6
TOP2B	DNA topoisomerase 2-beta
DKC1	H/ACA ribonucleoprotein complex subunit 4
PCCB	Propionyl-CoA carboxylase beta chain, mitochondrial
TUBA1B	Tubulin alpha-1B chain
PKM	Pyruvate kinase PKM
RPS8	40S ribosomal protein S8
GNL3	Guanine nucleotide-binding protein-like 3
EBNA1BP2	Probable rRNA-processing protein EBP2
FTSJ3	pre-rRNA processing protein FTSJ3
DHX9	ATP-dependent RNA helicase A
RPL7A	60S ribosomal protein L7a
H2AFX	Histone H2AX
HNRNPU	Heterogeneous nuclear ribonucleoprotein U
H1FX	Histone H1x
ACTB	Actin, cytoplasmic 1
ACTG1	Actin, cytoplasmic 2
RRS1	Ribosome biogenesis regulatory protein homolog
DSP	Desmoplakin
HSP90AA1	Heat shock protein HSP 90-alpha
KRT17	Keratin, type I cytoskeletal 17
AP3D1	AP-3 complex subunit delta-1
DDX21	Nucleolar RNA helicase 2
TTF1	Transcription termination factor 1

SLC25A5	ADP/ATP translocase 2
HIST1H2AJ	Histone H2A type 1-J
HIST1H2AH	Histone H2A type 1-H
H2AFJ	Histone H2A.J
HIST1H2AD	Histone H2A type 1-D
HIST1H2AG	Histone H2A type 1
HIST1H2AC	Histone H2A type 1-C
HIST3H2A	Histone H2A type 3
HIST1H2AB	Histone H2A type 1-B/E
H1F0	Histone H1.0
HIST1H1A	Histone H1.1
HSP90AB1	Heat shock protein HSP 90-beta
LARP7	La-related protein 7
DDX18	ATP-dependent RNA helicase DDX18
PARP1	Poly [ADP-ribose] polymerase 1
LYAR	Cell growth-regulating nucleolar protein
RPL18	60S ribosomal protein L18
JUP	Junction plakoglobin
MMTAG2	Multiple myeloma tumor-associated protein 2
GTPBP4	Nucleolar GTP-binding protein 1
KRT77	Keratin, type II cytoskeletal 1b
MTDH	Protein LYRIC
RPL11	60S ribosomal protein L11
SON	Protein SON
RPL4	60S ribosomal protein L4
SUPT16H	FACT complex subunit SPT16
VIM	Vimentin
RPF2	Ribosome production factor 2 homolog
GPATCH4	G patch domain-containing protein 4
PRSS1	Trypsin-1
TRY6	Putative trypsin-6
HIST2H2BF	Histone H2B type 2-F
HIST1H2BH	Histone H2B type 1-H
HIST1H2BL	Histone H2B type 1-L
HIST1H2BC	Histone H2B type 1-C/E/F/G/I
HIST1H2BM	Histone H2B type 1-M
HIST1H2BD	Histone H2B type 1-D
HIST1H2BN	Histone H2B type 1-N
HIST1H2BK	Histone H2B type 1-K
H2BFS	Histone H2B type F-S
HLCS	Biotin--protein ligase
HIST3H2BB	Histone H2B type 3-B
HIST1H2BO	Histone H2B type 1-O
HIST1H2BB	Histone H2B type 1-B
HIST1H2BJ	Histone H2B type 1-J
HIST2H2BE	Histone H2B type 2-E
HNRNPA1	Heterogeneous nuclear ribonucleoprotein A1
HSPA9	Stress-70 protein, mitochondrial
HNRNPH1	Heterogeneous nuclear ribonucleoprotein H
HIST1H4A	Histone H4
RPL23A	60S ribosomal protein L23a
KRT78	Keratin, type II cytoskeletal 78
HIST1H1B	Histone H1.5
HSPA5	78 kDa glucose-regulated protein
RBMX	RNA-binding motif protein, X chromosome
FAU	40S ribosomal protein S30
DSG1	Desmoglein-1
RPS27A	Ubiquitin-40S ribosomal protein S27a

RPL18A	60S ribosomal protein L18a
RPS16	40S ribosomal protein S16
PRPF4B	Serine/threonine-protein kinase PRP4 homolog
MYBBP1A	Myb-binding protein 1A
RPL7	60S ribosomal protein L7
RPL29	60S ribosomal protein L29
EEF1A1	Elongation factor 1-alpha 1
EEF1A1P5	Putative elongation factor 1-alpha-like 3
RPL14	60S ribosomal protein L14
HRNR	Hornerin
GAPDH	Glyceraldehyde-3-phosphate dehydrogenase
RBMX2	RNA-binding motif protein, X-linked 2
RPL15	60S ribosomal protein L15
HP1BP3	Heterochromatin protein 1-binding protein 3
RPS26	40S ribosomal protein S26
HNRNPC	Heterogeneous nuclear ribonucleoproteins C1/C2
H2AFY2	Core histone macro-H2A.2
SRRM2	Serine/arginine repetitive matrix protein 2
RPL8	60S ribosomal protein L8
RPS9	40S ribosomal protein S9
PRDX4	Peroxiredoxin-4
RPL27	60S ribosomal protein L27
PTBP1	Polypyrimidine tract-binding protein 1
PTPLAD1	Very-long-chain (3R)-3-hydroxyacyl-[acyl-carrier protein] dehydratase 3
KRI1	Protein KRI1 homolog
RPL19	60S ribosomal protein L19
RBM34	RNA-binding protein 34
VDAC1	Voltage-dependent anion-selective channel protein 1
CTTN	Src substrate cortactin
HNRNPK	Heterogeneous nuclear ribonucleoprotein K
RRP1B	Ribosomal RNA processing protein 1 homolog B
RPL27A	60S ribosomal protein L27a
RPL13	60S ribosomal protein L13
SNRPD1	Small nuclear ribonucleoprotein Sm D1
SOX1	Transcription factor SOX-1
LDHA	L-lactate dehydrogenase A chain
RPL31	60S ribosomal protein L31
RPL22	60S ribosomal protein L22
EIF5B	Eukaryotic translation initiation factor 5B
PFN1	Profilin-1
SRP72	Signal recognition particle subunit SRP72
DDX3X	ATP-dependent RNA helicase DDX3X
GNL2	Nucleolar GTP-binding protein 2
VDAC2	Voltage-dependent anion-selective channel protein 2
RPL36AL	60S ribosomal protein L36a-like
MAP1B	Microtubule-associated protein 1B
HIST1H3A	Histone H3.1
RPL13A	60S ribosomal protein L13a
SRSF11	Serine/arginine-rich splicing factor 11
RPL24	60S ribosomal protein L24
RPL7L1	60S ribosomal protein L7-like 1
DEK	Protein DEK
RPL3	60S ribosomal protein L3
FLG2	Filaggrin-2
SRRM1	Serine/arginine repetitive matrix protein 1
YWHAE	14-3-3 protein epsilon
SURF6	Surfeit locus protein 6
PPIA	Peptidyl-prolyl cis-trans isomerase A

HNRNPA2B1	Heterogeneous nuclear ribonucleoproteins A2/B1
CDSN	Corneodesmosin
WDR36	WD repeat-containing protein 36
CCDC71	Coiled-coil domain-containing protein 71
GNB2L1	Guanine nucleotide-binding protein subunit beta-2-like 1
SRSF7	Serine/arginine-rich splicing factor 7
RPL34	60S ribosomal protein L34
RBBP6	E3 ubiquitin-protein ligase RBBP6
FUS	RNA-binding protein FUS
ASPM	Abnormal spindle-like microcephaly-associated protein
USP42	Ubiquitin carboxyl-terminal hydrolase 42
RPL39P5	Putative 60S ribosomal protein L39-like 5
RPL39	60S ribosomal protein L39
RPS25	40S ribosomal protein S25
PHGDH	D-3-phosphoglycerate dehydrogenase
KTN1	Kinectin
TNFRSF1B	Tumor necrosis factor receptor superfamily member 1B
LLPH	Protein LLP homolog
RPS24	40S ribosomal protein S24
RPL10A	60S ribosomal protein L10a
CDK13	Cyclin-dependent kinase 13
MECP2	Methyl-CpG-binding protein 2
SLAIN2	SLAIN motif-containing protein 2
RPL5	60S ribosomal protein L5
ZCCHC17	Nucleolar protein of 40 kDa
ARGLU1	Arginine and glutamate-rich protein 1

Supplementary Table S1B. Proteins appearing in three MS replicates of negative control – NLS-BioID2

Gene Name	Detailed Description
KRT9	Keratin, type I cytoskeletal 9
KRT1	Keratin, type II cytoskeletal 1
KRT10	Keratin, type I cytoskeletal 10
KRT2	Keratin, type II cytoskeletal 2 epidermal
MKI67	Antigen KI-67
TCOF1	Treacle protein
NAT10	N-acetyltransferase 10
NOL6	Nucleolar protein 6
NCL	Nucleolin
RBBP6	E3 ubiquitin-protein ligase RBBP6
KRT14	Keratin, type I cytoskeletal 14
BRD2	Bromodomain-containing protein 2
NONO	Non-POU domain-containing octamer-binding protein
KRT6A	Keratin, type II cytoskeletal 6A
KRT5	Keratin, type II cytoskeletal 5
KNOP1	Lysine-rich nucleolar protein 1
KRT6C	Keratin, type II cytoskeletal 6C
HNRNPA2B1	Heterogeneous nuclear ribonucleoproteins A2/B1
TOP2A	DNA topoisomerase 2-alpha
PARP1	Poly [ADP-ribose] polymerase 1
FLNA	Filamin-A
TOP2B	DNA topoisomerase 2-beta
TMPO	Lamina-associated polypeptide 2, isoforms beta/gamma
KRT6B	Keratin, type II cytoskeletal 6B
RSL1D1	Ribosomal L1 domain-containing protein 1
NOP2	Putative ribosomal RNA methyltransferase NOP2
KRT16	Keratin, type I cytoskeletal 16
DDX3X	ATP-dependent RNA helicase DDX3X
DDX21	Nucleolar RNA helicase 2
PHIP	PH-interacting protein
HNRNPA1	Heterogeneous nuclear ribonucleoprotein A1
RBM34	RNA-binding protein 34
HNRNPK	Heterogeneous nuclear ribonucleoprotein K
DSP	Desmoplakin
FTSJ3	pre-rRNA processing protein FTSJ3
HRNR	Hornerin
DDX50	ATP-dependent RNA helicase DDX50
MYBBP1A	Myb-binding protein 1A
SON	Protein SON
NPM1	Nucleophosmin
GPATCH4	G patch domain-containing protein 4
RRP1B	Ribosomal RNA processing protein 1 homolog B
HNRNPR	Heterogeneous nuclear ribonucleoprotein R
NOP58	Nucleolar protein 58
SFPQ	Splicing factor, proline- and glutamine-rich
PRPF4B	Serine/threonine-protein kinase PRP4 homolog
TAGLN2	Transgelin-2
NIFK	MKI67 FHA domain-interacting nucleolar phosphoprotein
KRT17	Keratin, type I cytoskeletal 17
PPP1CC	Serine/threonine-protein phosphatase PP1-gamma catalytic subunit
EBNA1BP2	Probable rRNA-processing protein EBP2
HIST2H2AB	Histone H2A type 2-B

H2AFY	Core histone macro-H2A.1
TPX2	Targeting protein for Xklp2
DDX18	ATP-dependent RNA helicase DDX18
AHCTF1	Protein ELYS
DDX17	Probable ATP-dependent RNA helicase DDX17
RRBP1	Ribosome-binding protein 1
KRI1	Protein KRI1 homolog
DHX15	Putative pre-mRNA-splicing factor ATP-dependent RNA helicase DHX15
NOLC1	Nucleolar and coiled-body phosphoprotein 1
TTF1	Transcription termination factor 1
PPM1G	Protein phosphatase 1G
ANXA2	Annexin A2
NSFL1C	NSFL1 cofactor p47
THOC2	THO complex subunit 2
DNAJB1	DnaJ homolog subfamily B member 1
BRD3	Bromodomain-containing protein 3
GNL2	Nucleolar GTP-binding protein 2
SF3B1	Splicing factor 3B subunit 1
BUD13	BUD13 homolog
HNRNPU	Heterogeneous nuclear ribonucleoprotein U
SRRM1	Serine/arginine repetitive matrix protein 1
HIST1H2AJ	Histone H2A type 1-J
HIST1H2AH	Histone H2A type 1-H
HIST1H2AD	Histone H2A type 1-D
HIST1H2AG	Histone H2A type 1
HIST1H2AC	Histone H2A type 1-C
HIST3H2A	Histone H2A type 3
HIST1H2AB	Histone H2A type 1-B/E
H2AFJ	Histone H2A.J
CCDC43	Coiled-coil domain-containing protein 43
WDR70	WD repeat-containing protein 70
LYAR	Cell growth-regulating nucleolar protein
WDR46	WD repeat-containing protein 46
PCMT1	Protein-L-isoaspartate(D-aspartate) O-methyltransferase
DDX42	ATP-dependent RNA helicase DDX42
FHL1	Four and a half LIM domains protein 1
CEBPZ	CCAAT/enhancer-binding protein zeta
HMGXB4	HMG domain-containing protein 4
DDX55	ATP-dependent RNA helicase DDX55
DKC1	H/ACA ribonucleoprotein complex subunit 4
JUP	Junction plakoglobin
GPKOW	G patch domain and KOW motifs-containing protein
SF3B2	Splicing factor 3B subunit 2
ZC3H18	Zinc finger CCCH domain-containing protein 18
DSG1	Desmoglein-1
SART1	U4/U6.U5 tri-snRNP-associated protein 1
TAF3	Transcription initiation factor TFIID subunit 3
SNW1	SNW domain-containing protein 1
KRT78	Keratin, type II cytoskeletal 78
CFDP1	Craniofacial development protein 1
MTDH	Protein LYRIC
HNRNPA3	Heterogeneous nuclear ribonucleoprotein A3
UBE2M	NEDD8-conjugating enzyme Ubc12
U2SURP	U2 snRNP-associated SURP motif-containing protein
TXNL1	Thioredoxin-like protein 1
U2AF2	Splicing factor U2AF 65 kDa subunit
FUS	RNA-binding protein FUS

KRT77	Keratin, type II cytoskeletal 1b
CBX5	Chromobox protein homolog 5
NACA	Nascent polypeptide-associated complex subunit alpha
SRRM2	Serine/arginine repetitive matrix protein 2
FRG1	Protein FRG1
MDC1	Mediator of DNA damage checkpoint protein 1
LARP7	La-related protein 7
PSPC1	Paraspeckle component 1
DDX24	ATP-dependent RNA helicase DDX24
HIST1H2BC	Histone H2B type 1-C/E/F/G/I
HIST2H2BF	Histone H2B type 2-F
HIST1H2BM	Histone H2B type 1-M
HIST1H2BD	Histone H2B type 1-D
HIST1H2BN	Histone H2B type 1-N
HIST1H2BL	Histone H2B type 1-L
HIST1H2BH	Histone H2B type 1-H
HIST1H2BK	Histone H2B type 1-K
H2BFS	Histone H2B type F-S
CXorf56	UPF0428 protein Cxorf56
CTTN	Src substrate cortactin
PINX1	PIN2/TERF1-interacting telomerase inhibitor 1
PDS5A	Sister chromatid cohesion protein PDS5 homolog A
TCEB3	Transcription elongation factor B polypeptide 3
SCAF1	Splicing factor, arginine/serine-rich 19
NOP56	Nucleolar protein 56
H2AFV	Histone H2A.V
H2AFZ	Histone H2A.Z
ZNF711	Zinc finger protein 711
BMS1	Ribosome biogenesis protein BMS1 homolog
PHF2	Lysine-specific demethylase PHF2
HIST1H2BO	Histone H2B type 1-O
HIST1H2BB	Histone H2B type 1-B
HIST1H2BJ	Histone H2B type 1-J
HIST2H2BE	Histone H2B type 2-E
TARDBP	TAR DNA-binding protein 43
TOP1	DNA topoisomerase 1
DNTTIP2	Deoxynucleotidyltransferase terminal-interacting protein 2
BBX	HMG box transcription factor BBX
BAZ1B	Tyrosine-protein kinase BAZ1B
SUPT16H	FACT complex subunit SPT16
HN1	Hematological and neurological expressed 1 protein
EIF5B	Eukaryotic translation initiation factor 5B
MECP2	Methyl-CpG-binding protein 2
DHX9	ATP-dependent RNA helicase A
USP39	U4/U6.U5 tri-snRNP-associated protein 2
GPALPP1	GPALPP motifs-containing protein 1
RSBN1L	Round spermatid basic protein 1-like protein
KIN	DNA/RNA-binding protein KIN17
HNRNPA0	Heterogeneous nuclear ribonucleoprotein A0
WIBG	Partner of Y14 and mago
C12orf43	Uncharacterized protein C12orf43
KRT15	Keratin, type I cytoskeletal 15
TCERG1	Transcription elongation regulator 1
MED1	Mediator of RNA polymerase II transcription subunit 1
RBM25	RNA-binding protein 25
GNL3	Guanine nucleotide-binding protein-like 3
CSNK1A1	Casein kinase I isoform alpha

HIST1H4A	Histone H4
SSRP1	FACT complex subunit SSRP1
EXOSC10	Exosome component 10
KRT13	Keratin, type I cytoskeletal 13
SAFB	Scaffold attachment factor B1
THRAP3	Thyroid hormone receptor-associated protein 3
PCNP	PEST proteolytic signal-containing nuclear protein
DDX46	Probable ATP-dependent RNA helicase DDX46
KRT4	Keratin, type II cytoskeletal 4
PSIP1	PC4 and SFRS1-interacting protein
DHX29	ATP-dependent RNA helicase DHX29
ABCF1	ATP-binding cassette sub-family F member 1
H1F0	Histone H1.0
ZC3H11A	Zinc finger CCCH domain-containing protein 11A
NAP1L1	Nucleosome assembly protein 1-like 1
CD2BP2	CD2 antigen cytoplasmic tail-binding protein 2
HIST1H1E	Histone H1.4
RBMX	RNA-binding motif protein, X chromosome
SMCHD1	Structural maintenance of chromosomes flexible hinge domain-containing protein 1
SMAP	Small acidic protein
PPIL4	Peptidyl-prolyl cis-trans isomerase-like 4
RPL4	60S ribosomal protein L4
RPL6	60S ribosomal protein L6
EIF5A	Eukaryotic translation initiation factor 5A-1
HDGFRP2	Hepatoma-derived growth factor-related protein 2
TUBA1B	Tubulin alpha-1B chain
EXOSC9	Exosome complex component RRP45
CBX3	Chromobox protein homolog 3
CCDC137	Coiled-coil domain-containing protein 137
LRRC59	Leucine-rich repeat-containing protein 59
ANKRD11	Ankyrin repeat domain-containing protein 11
DEK	Protein DEK
C1orf52	UPF0690 protein C1orf52
LTV1	Protein LTV1 homolog
PC	Pyruvate carboxylase, mitochondrial
KRT80	Keratin, type II cytoskeletal 80
SUGP2	SURP and G-patch domain-containing protein 2
HNRNPDL	Heterogeneous nuclear ribonucleoprotein D-like
UTP14A	U3 small nucleolar RNA-associated protein 14 homolog A
COIL	Coilin
HP1BP3	Heterochromatin protein 1-binding protein 3
PRDX1	Peroxiredoxin-1
BRD7	Bromodomain-containing protein 7
FLG2	Filaggrin-2
AP3D1	AP-3 complex subunit delta-1
C19orf43	Uncharacterized protein C19orf43
YY1	Transcriptional repressor protein YY1
LUC7L3	Luc7-like protein 3
RPL14	60S ribosomal protein L14
DDX27	Probable ATP-dependent RNA helicase DDX27
SAP30BP	SAP30-binding protein
TAF2	Transcription initiation factor TFIID subunit 2
PDAP1	28 kDa heat- and acid-stable phosphoprotein
ZNF638	Zinc finger protein 638
HNRNPF	Heterogeneous nuclear ribonucleoprotein F
MMTAG2	Multiple myeloma tumor-associated protein 2

ANXA1	Annexin A1
PAPD5	PAP-associated domain-containing protein 5
TRIM28	Transcription intermediary factor 1-beta
ADNP	Activity-dependent neuroprotector homeobox protein
NOL8	Nucleolar protein 8
SBSN	Suprabasin
LBR	Lamin-B receptor
ACTG1	Actin, cytoplasmic 2
ACTB	Actin, cytoplasmic 1
BCLAF1	Bcl-2-associated transcription factor 1
POLR1E	DNA-directed RNA polymerase I subunit RPA49
HNRNPD	Heterogeneous nuclear ribonucleoprotein D0
GAPDH	Glyceraldehyde-3-phosphate dehydrogenase
NUP50	Nuclear pore complex protein Nup50
RANGAP1	Ran GTPase-activating protein 1
CCDC86	Coiled-coil domain-containing protein 86
RPS8	40S ribosomal protein S8
PRSS1	Trypsin-1
TRY6	Putative trypsin-6
PKP1	Plakophilin-1
STMN1	Stathmin
NOC3L	Nucleolar complex protein 3 homolog
ACIN1	Apoptotic chromatin condensation inducer in the nucleus
UBA52	Ubiquitin-60S ribosomal protein L40
RPS27A	Ubiquitin-40S ribosomal protein S27a
UBB	Polyubiquitin-B
UBC	Polyubiquitin-C
EEF1A1P5	Putative elongation factor 1-alpha-like 3
EEF1A1	Elongation factor 1-alpha 1
RPS10	40S ribosomal protein S10
DDX52	Probable ATP-dependent RNA helicase DDX52
ZRANB2	Zinc finger Ran-binding domain-containing protein 2
NPM3	Nucleoplasmin-3
HNRNPH1	Heterogeneous nuclear ribonucleoprotein H
SUB1	Activated RNA polymerase II transcriptional coactivator p15
NUCKS1	Nuclear ubiquitous casein and cyclin-dependent kinase substrate 1
HNRNPC	Heterogeneous nuclear ribonucleoproteins C1/C2
NHP2	H/ACA ribonucleoprotein complex subunit 2
TGM3	Protein-glutamine gamma-glutamyltransferase E
KRT24	Keratin, type I cytoskeletal 24
APEX1	DNA-(apurinic or apyrimidinic site) lyase
VIM	Vimentin
HMGA1	High mobility group protein HMG-I/HMG-Y
KRT84	Keratin, type II cuticular Hb4
ZCCHC17	Nucleolar protein of 40 kDa
HSPA5	78 kDa glucose-regulated protein
PCBP2	Poly(rC)-binding protein 2
HMGB3	High mobility group protein B3
HNRNPAB	Heterogeneous nuclear ribonucleoprotein A/B
C1orf131	Uncharacterized protein C1orf131
SURF6	Surfeit locus protein 6
RPL18	60S ribosomal protein L18
CWC27	Peptidyl-prolyl cis-trans isomerase CWC27 homolog
RP9	Retinitis pigmentosa 9 protein
RPL29	60S ribosomal protein L29
CAT	Catalase
CASP14	Caspase-14

DSC1	Desmocollin-1
FBL	rRNA 2'-O-methyltransferase fibrillarin
DCD	Dermcidin
KRT12	Keratin, type I cytoskeletal 12
RPL23A	60S ribosomal protein L23a
H1FX	Histone H1x
HSPA1A	Heat shock 70 kDa protein 1A/1B
MAP1B	Microtubule-associated protein 1B
ILKAP	Integrin-linked kinase-associated serine/threonine phosphatase 2C
CDK11A	Cyclin-dependent kinase 11A
CDK11B	Cyclin-dependent kinase 11B
RPL19	60S ribosomal protein L19
PRDX2	Peroxiredoxin-2
TUBB	Tubulin beta chain
RIF1	Telomere-associated protein RIF1
CDSN	Corneodesmosin
CHD4	Chromodomain-helicase-DNA-binding protein 4
BUD31	Protein BUD31 homolog
S100A8	Protein S100-A8
PARN	Poly(A)-specific ribonuclease PARN
TUBB4B	Tubulin beta-4B chain
INTS12	Integrator complex subunit 12
CDC5L	Cell division cycle 5-like protein
ANP32C	Acidic leucine-rich nuclear phosphoprotein 32 family member C
ANP32A	Acidic leucine-rich nuclear phosphoprotein 32 family member A
SSB	Lupus La protein
RPS25	40S ribosomal protein S25
HSPA8	Heat shock cognate 71 kDa protein
PPIA	Peptidyl-prolyl cis-trans isomerase A
GTPBP4	Nucleolar GTP-binding protein 1
NGDN	Neuroguidin
RPL24	60S ribosomal protein L24
HSP90AB1	Heat shock protein HSP 90-beta
MLLT6	Protein AF-17
GAR1	H/ACA ribonucleoprotein complex subunit 1
RRP15	RRP15-like protein
EDF1	Endothelial differentiation-related factor 1
SRSF7	Serine/arginine-rich splicing factor 7
TXN	Thioredoxin
FAU	40S ribosomal protein S30
C11orf57	Uncharacterized protein C11orf57
PFN1	Profilin-1
GK3P	Putative glycerol kinase 3
GK	Glycerol kinase
TGM1	Protein-glutamine gamma-glutamyltransferase K
H3F3A	Histone H3.3
HIST2H3A	Histone H3.2
HIST1H3A	Histone H3.1
HIST3H3	Histone H3.1t
H3F3C	Histone H3.3C
SHOX2	Short stature homeobox protein 2
CBX1	Chromobox protein homolog 1
POF1B	Protein POF1B
TUFM	Elongation factor Tu, mitochondrial
HDAC1	Histone deacetylase 1
LLPH	Protein LLP homolog
PIAS2	E3 SUMO-protein ligase PIAS2

ALB	Serum albumin
GSDMA	Gasdermin-A
XP32	Skin-specific protein 32
ATP5A1	ATP synthase subunit alpha, mitochondrial
HAL	Histidine ammonia-lyase
ATXN3	Ataxin-3
PIN4	Peptidyl-prolyl cis-trans isomerase NIMA-interacting 4
IGF2BP1	Insulin-like growth factor 2 mRNA-binding protein 1
CHAMP1	Chromosome alignment-maintaining phosphoprotein 1
PHF6	PHD finger protein 6
SERPINB12	Serpin B12
PKM	Pyruvate kinase PKM
NOP14	Nucleolar protein 14
EIF3C	Eukaryotic translation initiation factor 3 subunit C
EIF3CL	Eukaryotic translation initiation factor 3 subunit C-like protein
LMNA	Prelamin-A/C
USP31	Ubiquitin carboxyl-terminal hydrolase 31
RPS16	40S ribosomal protein S16
DCP2	m7GpppN-mRNA hydrolase
POU4F2	POU domain, class 4, transcription factor 2
DNAH17	Dynein heavy chain 17, axonemal

Supplementary Table S2A. BioID2 candidates exclusive to Ku70 S155A using first filtering approach, appearing in three MS replicates.

Gene Name	Detailed Description
RPAP3	RNA polymerase II-associated protein 3
NUDCD2	NudC domain-containing protein 2
C20orf27	UPF0687 protein C20orf27

Supplementary Table S2B. BioID2 candidates exclusive to Ku70 S155D using first filtering approach, that appearing in three MS replicates.

Gene Name	Detailed Description
WHSC1	Histone-lysine N-methyltransferase NSD2
CHD3	Chromodomain-helicase-DNA-binding protein 3
ZFR	Zinc finger RNA-binding protein
MPG	DNA-3-methyladenine glycosylase
HNRNPUL1	Heterogeneous nuclear ribonucleoprotein U-like protein 1
CBX8	Chromobox protein homolog 8
DDX5	Probable ATP-dependent RNA helicase DDX5
ARG1	Arginase-1
ZNF512	Zinc finger protein 512
PHF8	Histone lysine demethylase PHF8
ZNF362	Zinc finger protein 362
DACH1	Dachshund homolog 1
C5orf24	UPF0461 protein C5orf24
YWHAZ	14-3-3 protein zeta/delta
C10orf12	Uncharacterized protein C10orf12
CTCF	Transcriptional repressor CTCF
ATRX	Transcriptional regulator ATRX
BLM	Bloom syndrome protein
SCML2	Sex comb on midleg-like protein 2
YLPM1	YLP motif-containing protein 1
ATP5O	ATP synthase subunit O, mitochondrial
USP36	Ubiquitin carboxyl-terminal hydrolase 36
ZNF292	Zinc finger protein 292
PDCD11	Protein RRP5 homolog
SENP3	Sentrin-specific protease 3
ATAD5	ATPase family AAA domain-containing protein 5
WDR18	WD repeat-containing protein 18
CWF19L2	CWF19-like protein 2
CDCA8	Borealin
BPTF	Nucleosome-remodeling factor subunit BPTF
ATP1A1	Sodium/potassium-transporting ATPase subunit alpha-1
WRN	Werner syndrome ATP-dependent helicase
ZNF280D	Zinc finger protein 280D
SMARCA2	Probable global transcription activator SNF2L2
TFAP2A	Transcription factor AP-2-alpha
TRIP12	E3 ubiquitin-protein ligase TRIP12
MDH2	Malate dehydrogenase, mitochondrial
CAND1	Cullin-associated NEDD8-dissociated protein 1
CHERP	Calcium homeostasis endoplasmic reticulum protein
EWSR1	RNA-binding protein EWS
UBE2E1	Ubiquitin-conjugating enzyme E2 E1
MLLT1	Protein ENL
SLX4	Structure-specific endonuclease subunit SLX4
ZFP91	E3 ubiquitin-protein ligase ZFP91
TAGLN3	Transgelin-3
C1orf174	UPF0688 protein C1orf174
ZNF24	Zinc finger protein 24
RAB10	Ras-related protein Rab-10
POGZ	Pogo transposable element with ZNF domain
WIZ	Protein Wiz
MSH2	DNA mismatch repair protein Msh2
TEX10	Testis-expressed sequence 10 protein
RAI1	Retinoic acid-induced protein 1

CCAR2	Cell cycle and apoptosis regulator protein 2
KIF22	Kinesin-like protein KIF22
SRSF2	Serine/arginine-rich splicing factor 2
SRSF8	Serine/arginine-rich splicing factor 8
NSA2	Ribosome biogenesis protein NSA2 homolog

Supplementary Table S3A. SAINTexpress candidates scoring greater than or equal to 0.6 – wild-type Ku70-BioID2.

*1 = in BioGRID, 0 = not in BioGRID

Gene Name	SAINTexpress Score	BioGRID
ENO1	1	1
HSPD1	1	0
XRCC6	1	1
VDAC3	1	0
ARG1	1	0
RPS19	1	0
RPS18	1	0
MTHFD1	0.99	0
ATP5F1B	0.99	0
PA2G4	0.99	0
HSP90B1	0.98	0
PHGDH	0.98	0
SLC25A3	0.98	0
RPS4X	0.97	0
HNRNPM	0.97	0
HSPA9	0.96	0
PFN1	0.95	0
HSP90AB1	0.94	0
H2AC4	0.92	1
HAL	0.91	0
TGM1	0.9	0
UBA1	0.9	0
SERPINB12	0.9	0
RPL27A	0.89	0
DSC1	0.88	0
HIST1H2BB	0.86	0
PHB	0.85	1
H2AFV	0.85	0
TIMM50	0.81	0
HSP90AA1	0.81	0
VDAC1	0.8	1
PRKDC	0.79	1
H2BC4	0.79	0
LDHB	0.78	0
PHB2	0.73	1
KRT15	0.69	0
KRT8	0.67	0
NPEPPS	0.66	0
ALB	0.66	0
EEF2	0.66	1
H2AFY2	0.66	0
CANX	0.66	0
LYZ	0.66	0
CAND1	0.66	1
LDHA	0.66	0
CCT8	0.65	1
CCT3	0.65	1
ATP1A1	0.65	0
IGF2BP1	0.65	0
EEF1G	0.65	0
CLTC	0.65	1
EIF2S3	0.64	0
SRSF7	0.64	1
MAP1B	0.64	0
RPL36A	0.64	0
SPTBN1	0.64	0
CCT6A	0.63	0
TUBA1B	0.63	0
CKB	0.62	0

Supplementary Table S3B. SAINTexpress candidates scoring greater than or equal to 0.6 – Ku70 S155A-BioID2.

*1 = in BioGRID, 0 = not in BioGRID

Gene Name	SAINTexpress Score	BioGRID
CCT8	1	1
XRCC6	1	1
RPAP3	1	0
RUVBL2	1	0
NUDC	0.99	0
DVL2	0.99	0
PIH1D1	0.99	1
CACYBP	0.98	0
SSB	0.97	1
DNAJA2	0.97	0
HSPD1	0.97	0
DVL3	0.94	0
ENO1	0.91	1
SUGT1	0.88	0
CHORDC1	0.84	0
UBA1	0.83	0
HIST1H2BB	0.82	0
PFN1	0.75	0
H2BC4	0.75	0
HSP90AA1	0.69	0
FKBP4	0.67	0
RUVBL1	0.67	1
MAP1B	0.67	0
HSP90B1	0.66	0
EEF2	0.66	1
PPIA	0.66	0
RPA1	0.66	1
LDHA	0.66	0
STIP1	0.65	0
RAN	0.65	0
NUDCD2	0.65	0
IGF2BP1	0.65	0
MATR3	0.65	1
H2AFY2	0.65	0
MTA1	0.65	0
TUBA1A	0.65	0
CCT3	0.64	1
CCT4	0.64	0
CCT5	0.64	0
STUB1	0.64	0
PIAS2	0.64	1
RPS18	0.64	0
SNRPD1	0.63	0
TRIM28	0.61	1
CD2BP2	0.6	0

Supplementary Table S3C. SAINTexpress candidates scoring greater than or equal to 0.6 – Ku70 S155D-BioID2.

*1 = in BioGRID, 0 = not in BioGRID

Gene Name	SAINTexpress Score	BioGRID
CCT8	1	1
GTF2B	1	0
CHAMP1	1	1
MGA	1	1
RALY	1	1
CDCA2	1	0
EEF2	1	1
SF1	1	1
KHSRP	1	1
SSB	1	1
XRCC5	1	1
XRCC6	1	1
INTS1	1	0
MSH6	1	1
KMT2A	1	0
RPA1	1	1
SART3	1	1
LIN54	1	0
NUMA1	1	1
ZNF250	1	0
RPS19	1	0
RUVBL2	1	0
GDI2	0.99	0
RBM10	0.99	1
RIF1	0.99	1
CDK12	0.99	1
TRIP12	0.99	1
RAN	0.99	0
NSD1	0.99	0
ENO1	0.99	1
NSUN2	0.99	0
MATR3	0.99	1
HSPD1	0.99	0
CHD3	0.99	1
CHD4	0.99	1
PRDM16	0.99	0
NCOR1	0.99	1
WAPL	0.99	1
PIAS1	0.99	0
RPS18	0.99	0
MTA1	0.99	0
CHD8	0.99	1
GTF2F1	0.98	0
AHCTF1	0.98	1
DVL2	0.98	0
DVL3	0.98	0
SMCHD1	0.98	1

Gene Name	SAINTexpress Score	BioGRID
CCT5	0.97	0
SET	0.97	1
PIAS2	0.97	1
PFN1	0.96	0
UBA1	0.95	0
PCBP2	0.94	0
TRIM28	0.93	1
DDX42	0.92	1
BAZ1B	0.91	1
RTF1	0.91	1
MDC1	0.9	1
VDAC1	0.9	1
PCBP1	0.89	1
BCLAF1	0.89	1
VDAC2	0.88	0
DDX46	0.87	1
RPL27A	0.87	0
CDKN2AIP	0.87	0
UTP14A	0.86	1
RPL19	0.86	0
ZNF638	0.85	1
CKB	0.85	0
PHF2	0.84	0
ZNF512B	0.84	1
NSD2	0.84	0
TCERG1	0.83	1
NUP50	0.83	1
SAP30BP	0.82	1
KIFC1	0.82	0
RFC1	0.81	1
ZC3H4	0.8	1
MED1	0.8	1
CFDP1	0.8	1
RPS27A	0.79	0
INTS12	0.79	0
PTBP1	0.79	0
GPKOW	0.78	0
NBN	0.78	0
ELAVL1	0.76	0
ADNP	0.75	1
ILKAP	0.74	0
HSPA9	0.73	0
CWC27	0.73	0
PEBP1	0.72	0
ZC3H11A	0.71	1
PPM1G	0.71	1
SUGT1	0.7	0

Gene Name	SAINTexpress Score	BioGRID
EXOSC10	0.68	1
DHX38	0.68	1
ILF3	0.68	1
WRN	0.67	1
GTF2I	0.67	1
ZNF384	0.67	0
NR2C2	0.67	1
RAI1	0.67	0
NASP	0.67	1
BPTF	0.67	1
KIF23	0.67	1
KIF22	0.67	0
HDGF	0.67	1
YY1	0.67	1
LDHA	0.67	0
NUP153	0.67	1
RBL1	0.67	0
WIZ	0.67	1
HCFC1	0.67	1
HSPA4	0.66	0
H2AFY2	0.66	0
NELFA	0.66	0
CTBP2	0.66	1
PPP1R10	0.66	1
TBCB	0.66	0
PGD	0.66	0
XPC	0.66	1
CDCA8	0.66	0
RUVBL1	0.66	1
FASN	0.66	0
RPS3A	0.66	0
YWHAQ	0.66	0
PAPOLA	0.66	1
ZFR	0.66	1
RPS4X	0.66	0
ZMYND8	0.66	1
JUN	0.66	1
MAP1B	0.66	0
LMNB1	0.66	0
CUX1	0.66	0
RPS23	0.66	0
APEX1	0.66	1
NIPBL	0.66	0
PHF8	0.66	0
CANX	0.66	0
ZNF362	0.66	1
PABPC1	0.66	0
PHF21A	0.66	0
SRP14	0.66	0
ARG1	0.66	0
RBM27	0.66	1
HNRNPUL1	0.66	0
ORC2	0.66	1
ADAR	0.66	1

Gene Name	SAINTexpress Score	BioGRID
ANP32A	0.65	0
ZNF281	0.65	1
GTF3C4	0.65	1
CCT3	0.65	1
PSMD4	0.65	1
TCEA1	0.65	0
CHAF1B	0.65	0
WDR70	0.65	1
GMPS	0.65	0
POLD3	0.65	0
DIDO1	0.65	1
HNRNPM	0.65	0
HN1L	0.65	0
YWHAZ	0.65	1
YWHAE	0.65	0
SPEN	0.65	1
GLYR1	0.65	1
ATP5PO	0.65	0
RPLP0P6	0.65	0
PPIA	0.65	0
TFAP2A	0.65	1
CKAP2	0.65	0
ZNF292	0.65	0
HIST1H2BB	0.65	0
PIAS3	0.65	0
DACH1	0.65	1
HAT1	0.64	1
PRDX4	0.64	0
HSP90AA1	0.64	0
NVL	0.63	1
RPS2	0.63	0
CWF19L1	0.63	0
H2BC4	0.63	0
CHMP4B	0.62	0
PKM	0.62	0
SF3B1	0.62	1
GNB2L1	0.62	0
RPS24	0.62	0
HSPA5	0.61	1
CRKL	0.61	1
L3MBTL3	0.61	0
ARL6IP4	0.61	0
PRKDC	0.6	1
SREK1	0.6	0
PHB2	0.6	1

Uncropped blots

Figure 1a. Cell lines induced with or without 1 µg/mL doxycycline for 24 hours before analyzing by Western blotting. All Western blots depicted show 50 µg of whole cell extracts (WCE).

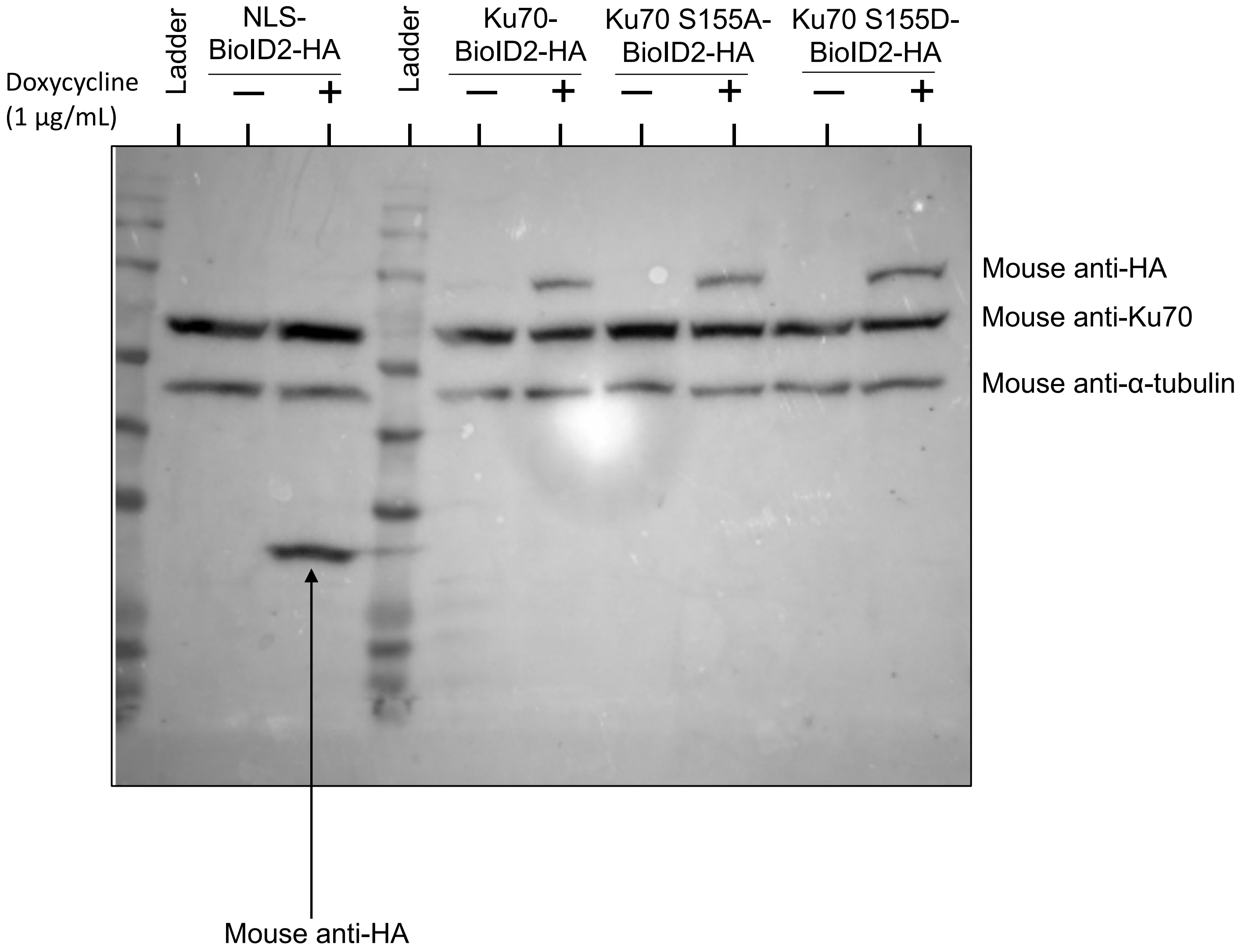

Note: Same blot probed with HA, Ku70, α-tubulin

Figure 1b. Time course following doxycycline induction (1 μg/mL) of Ku70 S155A-BioID2-HA and Ku70 S155D-BioID2-HA. All Western blots depicted show 50 μg of whole cell extracts (WCE).

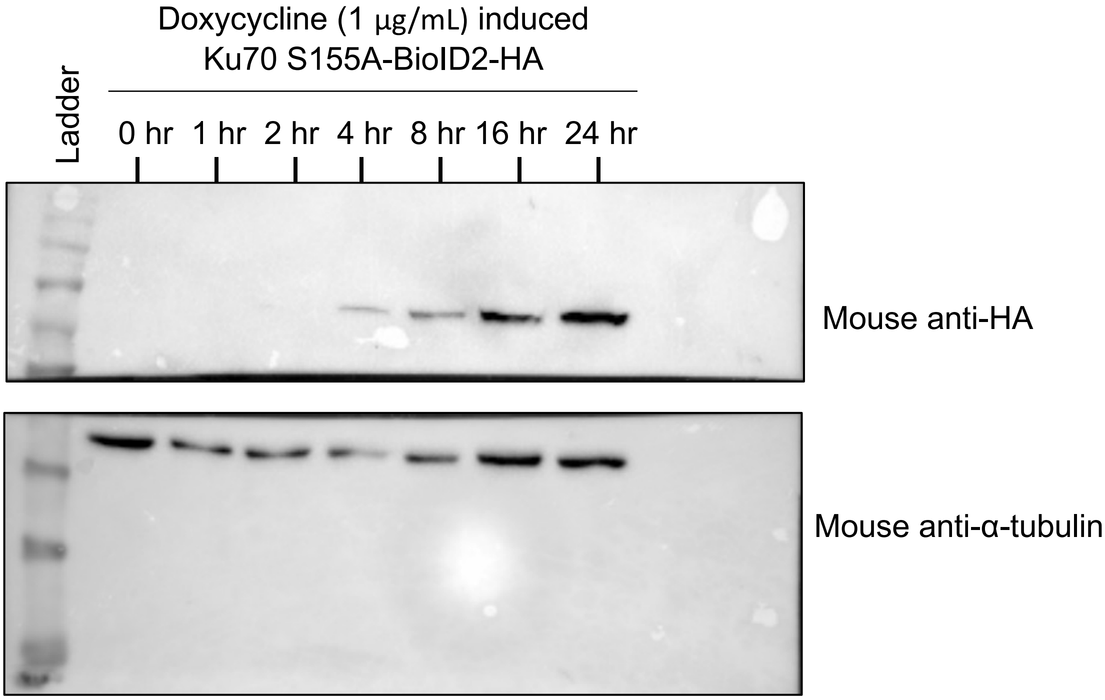

Note: Same blot probed with HA and α-tubulin

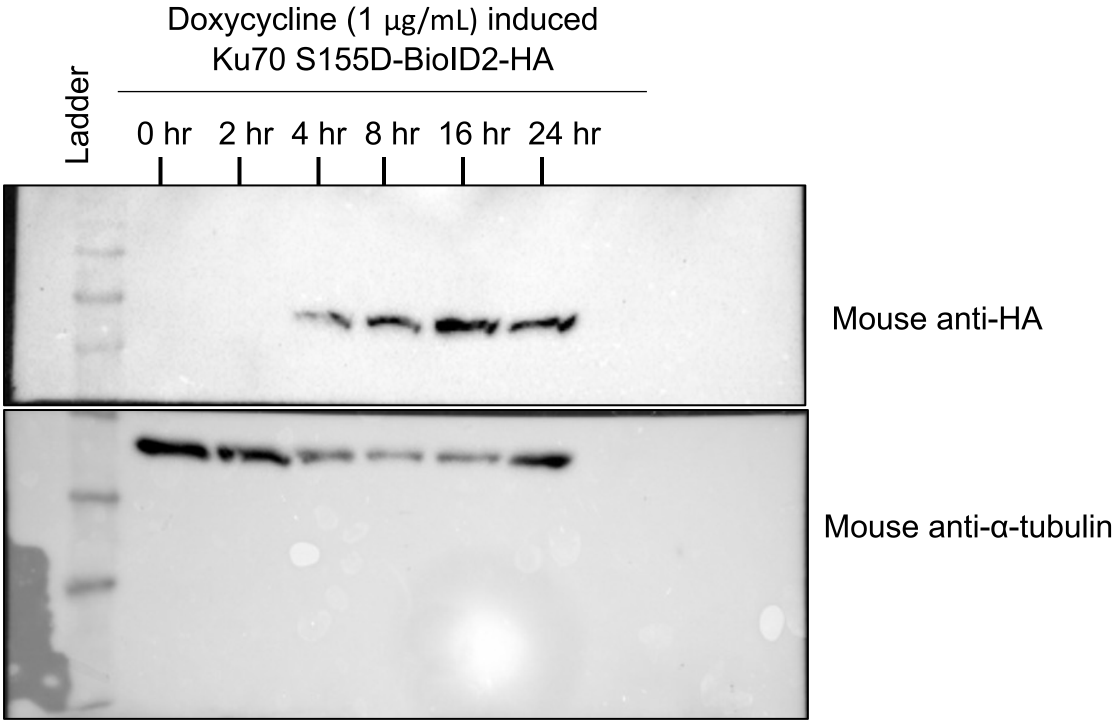

Note: Same blot probed with HA and α-tubulin

Figure 1d. Biotinylation of doxycycline (1 µg/mL)-induced stable cell lines. After 8 hr of doxycycline-induction, cell lines were supplemented with or without biotin (50 µM) for 24 hours prior to analysis by Western blotting (50 µg of WCE). Streptavidin blots are a separate gel loaded identically in parallel with same samples as HA, tubulin.

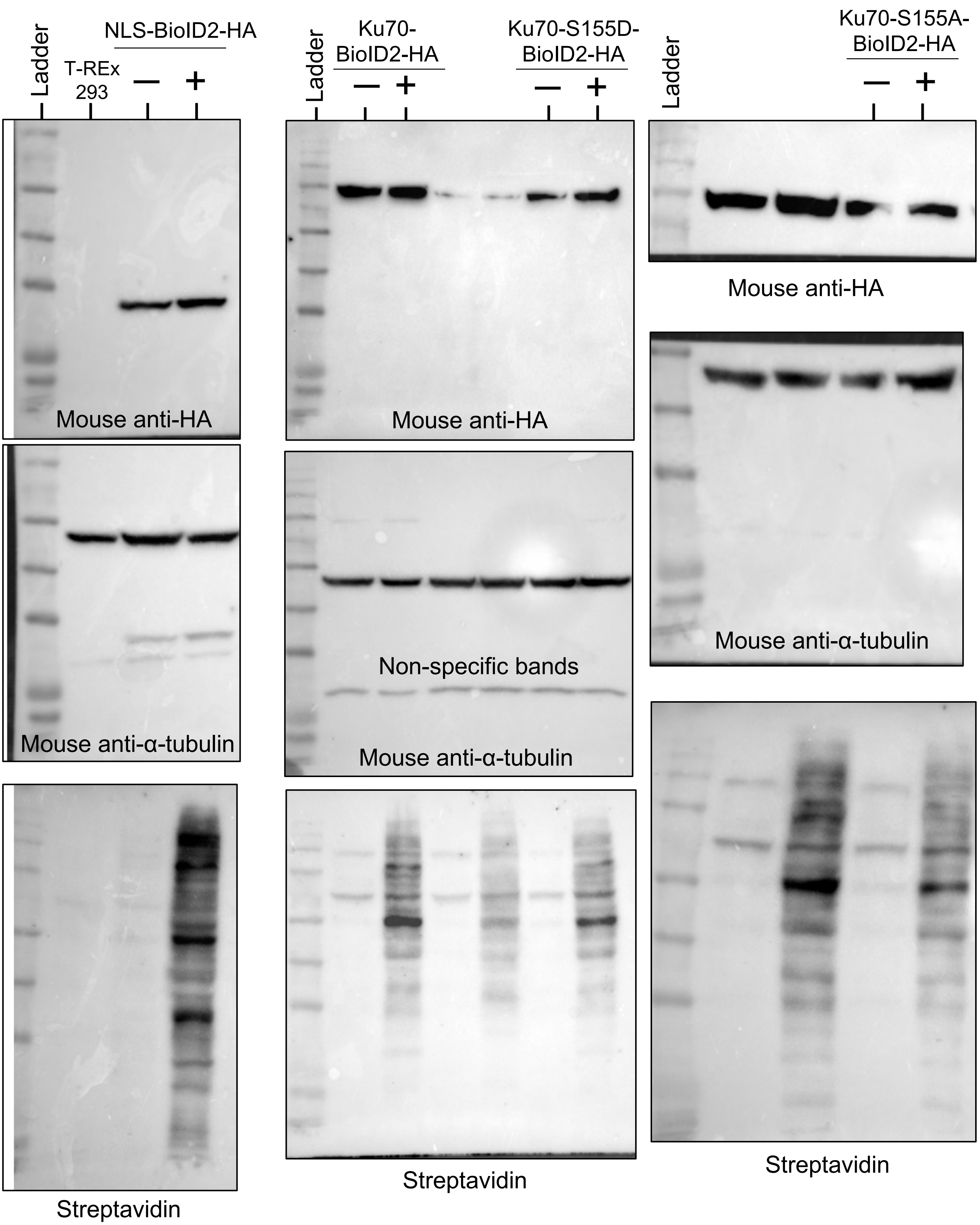

Figure 3a. Western blot of inducible stable cell lines with and without 24 hr of doxycycline. All Western blots depicted show 50 µg of whole cell extracts (WCE).

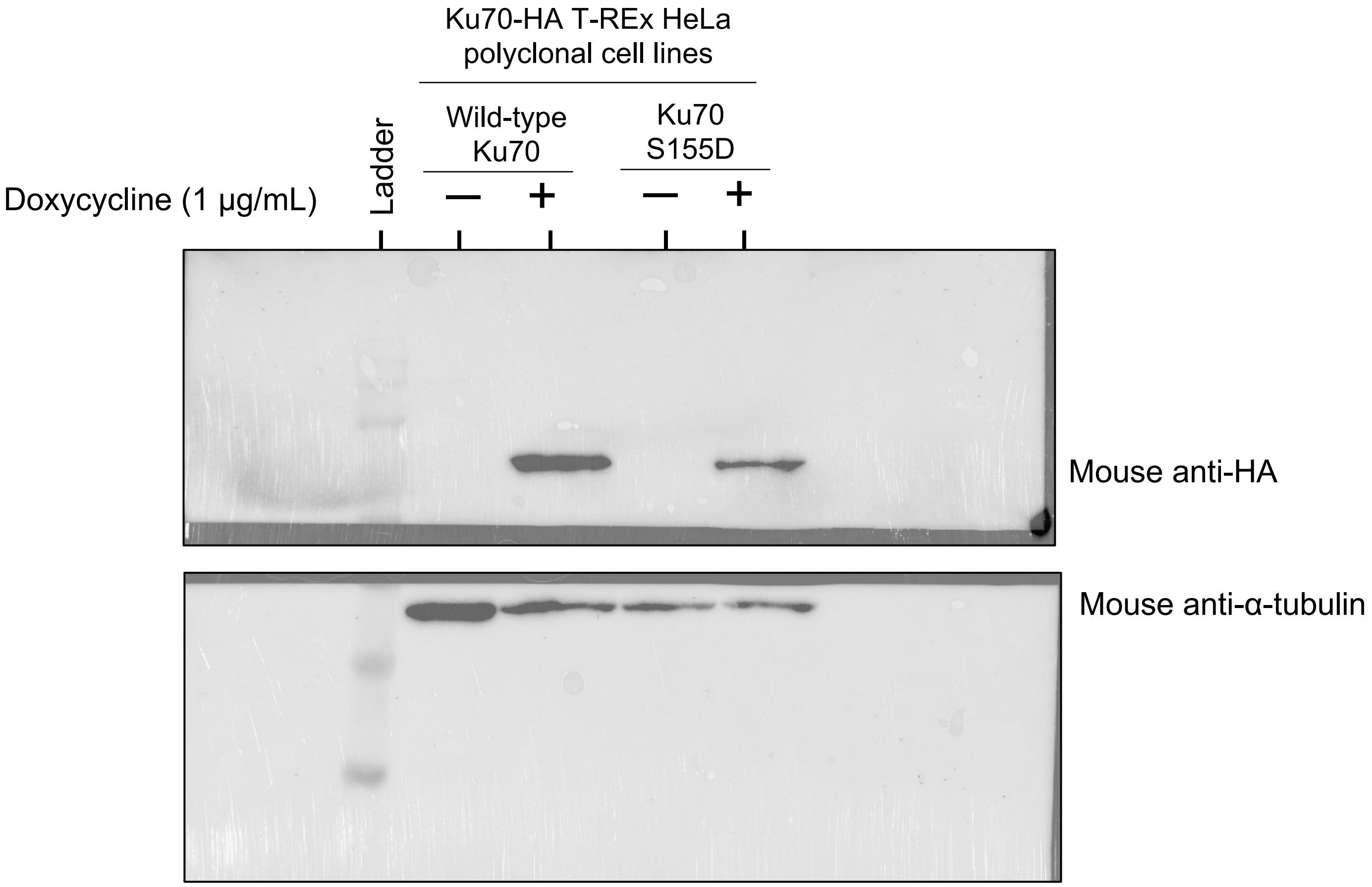

<u>Note</u>: Same blot probed with HA and α-tubulin

Figure 3b. Time course experiment showing the inducible expression of both polyclonal stable cell lines induced with doxycycline over a period of 24 hr. All Western blots depicted show 50 µg of whole cell extracts (WCE), with or without doxycycline (1 µg/mL).

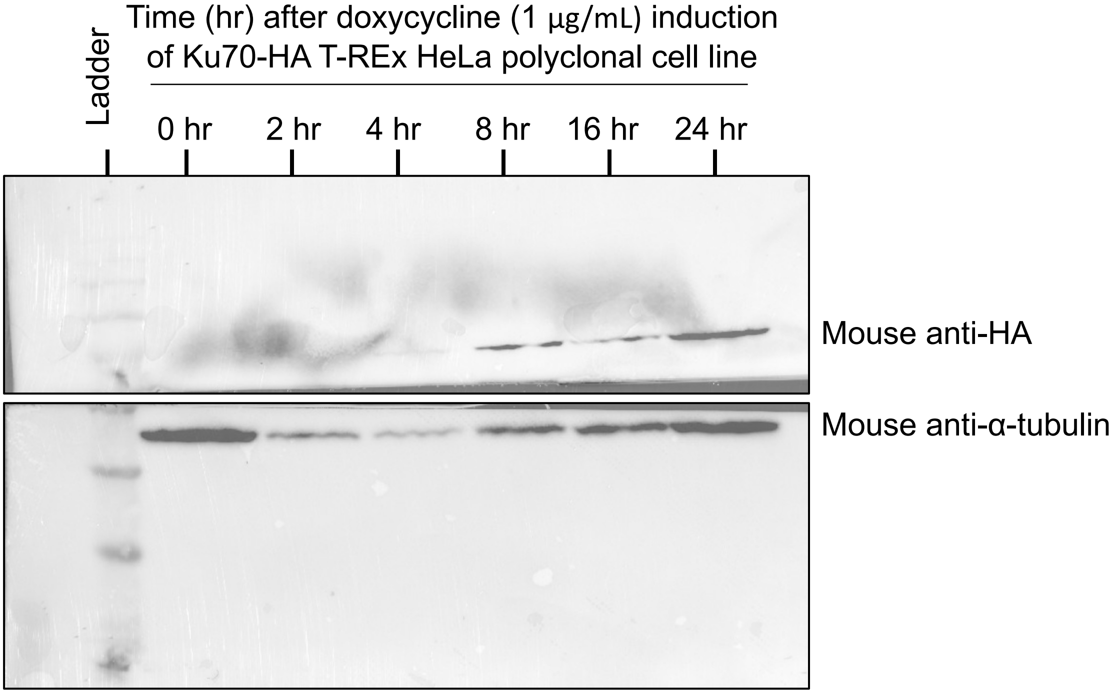

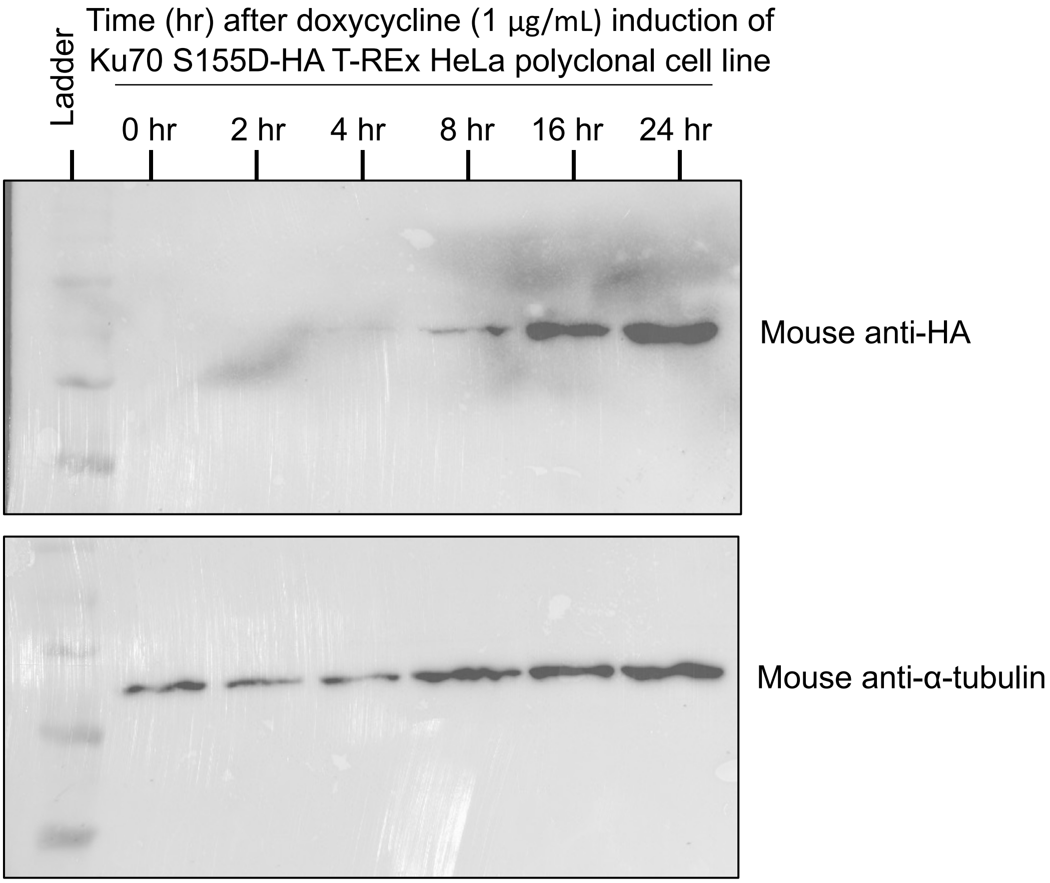

Figure 6. **Co-immunoprecipitation of Ku70 in HeLa cells treated with IR to produce DSBs:** Testing for an interaction between Ku70 and TRIP12. Co-immunoprecipitation conducted with 500µg of WCE in the presence or absence of 40gy IR.

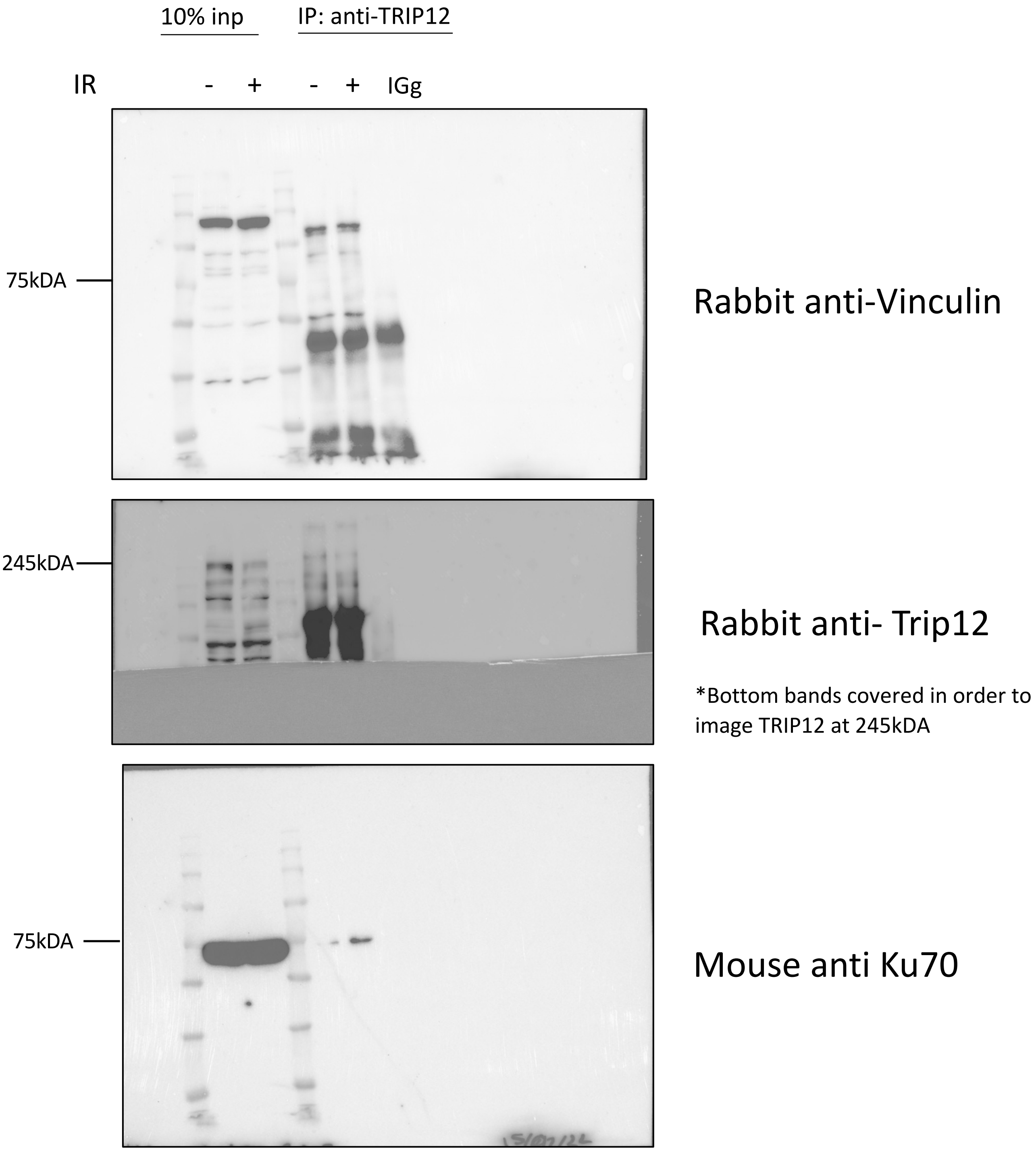

Figure S4. Doxycycline induction (1 μg/mL) of Ku70 S155A-HA

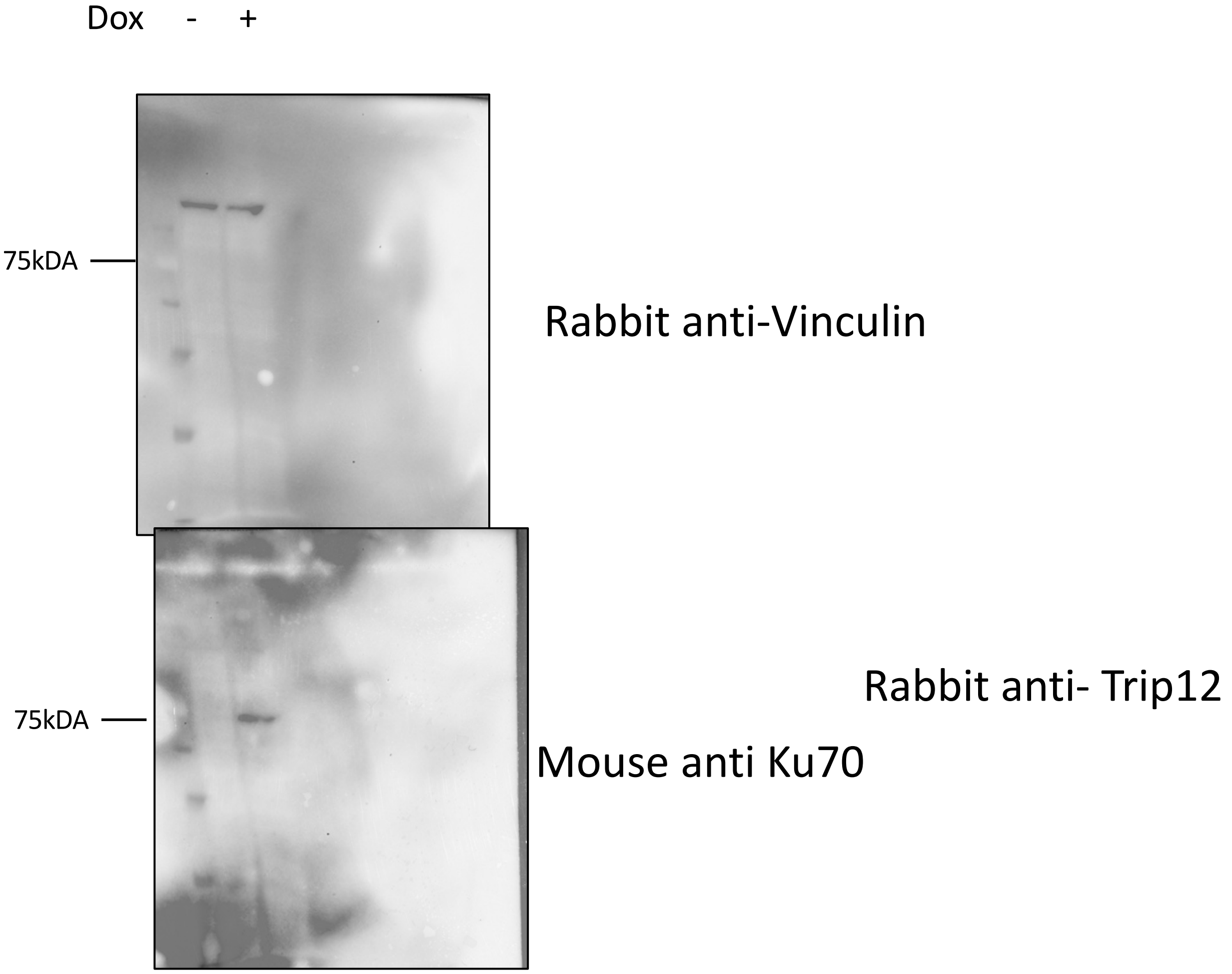

Supplement: Supplementary file 1 [file ijms-24-07041-s001.zip › ijms-2291553-supplementary.pdf]
